# Differences in Integron Cassette Excision Dynamics Shape a Trade-Off between Evolvability and Genetic Capacitance

**DOI:** 10.1128/mBio.02296-16

**Published:** 2017-03-28

**Authors:** Céline Loot, Aleksandra Nivina, Jean Cury, José Antonio Escudero, Magaly Ducos-Galand, David Bikard, Eduardo P. C. Rocha, Didier Mazel

**Affiliations:** a Unité de Plasticité du Génome Bactérien, Institut Pasteur, Paris, France; b Centre National de la Recherche Scientifique UMR 3525, Paris, France; c Université Paris Descartes, Sorbonne Paris Cité, Paris, France; d Microbial Evolutionary Genomics Unit, Institut Pasteur, Paris, France; University of British Columbia

**Keywords:** integron, *attC* sites, cassette dynamics, replication, site-specific recombination

## Abstract

Integrons ensure a rapid and “on demand” response to environmental stresses driving bacterial adaptation. They are able to capture, store, and reorder functional gene cassettes due to site-specific recombination catalyzed by their integrase. Integrons can be either sedentary and chromosomally located or mobile when they are associated with transposons and plasmids. They are respectively called sedentary chromosomal integrons (SCIs) and mobile integrons (MIs). MIs are key players in the dissemination of antibiotic resistance genes. Here, we used *in silico* and *in vivo* approaches to study cassette excision dynamics in MIs and SCIs. We show that the orientation of cassette arrays relative to replication influences *attC* site folding and cassette excision by placing the recombinogenic strands of *attC* sites on either the leading or lagging strand template. We also demonstrate that stability of *attC* sites and their propensity to form recombinogenic structures also regulate cassette excision. We observe that cassette excision dynamics driven by these factors differ between MIs and SCIs. Cassettes with high excision rates are more commonly found on MIs, which favors their dissemination relative to SCIs. This is especially true for SCIs carried in the *Vibrio* genus, where maintenance of large cassette arrays and vertical transmission are crucial to serve as a reservoir of adaptive functions. These results expand the repertoire of known processes regulating integron recombination that were previously established and demonstrate that, in terms of cassette dynamics, a subtle trade-off between evolvability and genetic capacitance has been established in bacteria.

## IMPORTANCE

The integron system confers upon bacteria a rapid adaptation capability in changing environments. Specifically, integrons are involved in the continuous emergence of bacteria resistant to almost all antibiotic treatments. The international situation is critical, and in 2050, the annual number of deaths caused by multiresistant bacteria could reach 10 million, exceeding the incidence of deaths related to cancer. It is crucial to increase our understanding of antibiotic resistance dissemination and therefore integron recombination dynamics to find new approaches to cope with the worldwide problem of multiresistance. Here, we studied the dynamics of recombination and dissemination of gene encoding cassettes carried on integrons. By combining *in silico* and *in vivo* analyses, we show that cassette excision is highly regulated by replication and by the intrinsic properties of cassette recombination sites. We also demonstrated differences in the dynamics of cassette recombination between mobile and sedentary chromosomal integrons (MIs and SCIs). For MIs, a high cassette recombination rate is favored and timed to conditions when generating diversity (upon which selection can act) allows for a rapid response to environmental conditions and stresses. In contrast, for SCIs, cassette excisions are less frequent, limiting cassette loss and ensuring a large pool of cassettes. We therefore confirm a role of SCIs as reservoirs of adaptive functions and demonstrate that the remarkable adaptive success of integron recombination system is due to its intricate regulation.

## INTRODUCTION

Antibiotics are essential to the success of modern medicine, but their efficacy has been impeded by the emergence of multiresistant bacteria. In 1989, integrons were identified as systems responsible for the dissemination of resistance genes among Gram-negative bacterial pathogens (1, 2), primarily due to their association with transposable elements and conjugative plasmids. The aforementioned systems were later named mobile integrons (MIs) as opposed to sedentary chromosomally located integrons (SCIs), which are found in Gram-negative bacteria from various environments and play a general role in bacterial evolution (3). The integron is a powerful genetic system that enables bacterial evolution by capturing, stockpiling, and reordering cassette-encoding proteins with potentially advantageous functions for adaptation to changing environments (antibiotic resistance, virulence, interaction with phages [4–8]).

**FIG 1:**
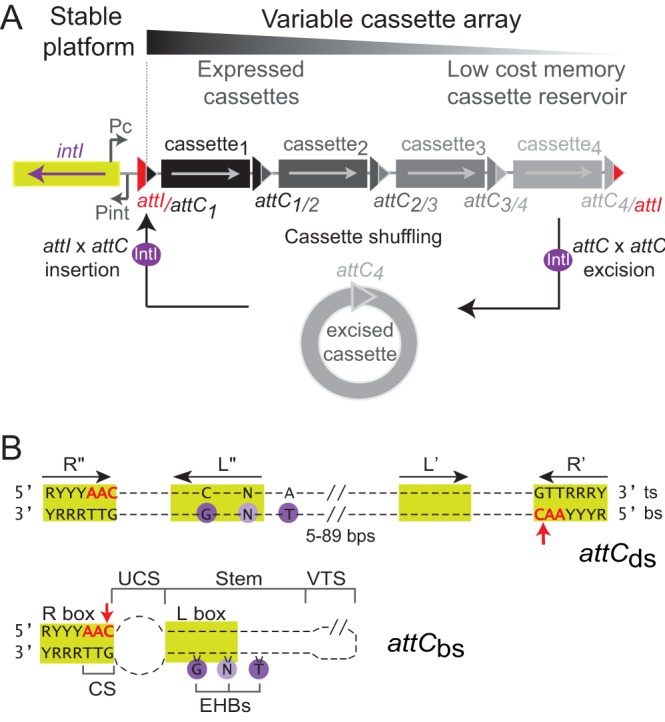
The integron system. (A) Organization of integrons. Functional platform composed of the *intI* gene encoding the integrase (green rectangle), the cassette promoter (P_C_) and the integrase promoter (Pint), as well as the primary *attI* recombination site (red triangle) are shown. Integrase (IntI; purple circle) catalyzes cassette excision (*attC* × *attC*) followed by insertion (*attI* × *attC*) of the excised cassette (gray circle). Hybrid *att* sites are indicated. Arrows inside the cassettes indicate the direction of their coding sequence (CDS), and the color intensity reflects the expression level of cassettes: only the first several cassettes of the array are expressed, while the subsequent ones can be seen as a low-cost cassette reservoir. (B) *attC* recombination sites. The double- and single-stranded *attC* sites (*attC*_ds_ and *attC*_bs_) are shown. Green boxes show putative IntI1 binding sites, and red arrows show the cleavage point. For the *attC*_ds_, inverted repeats (R’’, L”, L’, and R’) are indicated by black arrows. The conserved nucleotides are indicated, and violet circles show the conserved G nucleotide and the other bases, which constitute the extrahelical bases (EHBs) in folded *attC* sites (see *attC*_bs_). The top strand (ts) and bottom strand (bs) are marked. The structure of *attC*_bs _was determined by the RNAfold program from ViennaRNA 2 package (Materials and Methods). Structural features, namely, the unpaired central spacer (UCS), the EHBs, the stem, and the variable terminal structure (VTS), as well as the conserved sequence (CS), are indicated. R, purine; Y, pyrimidine; N, any base.

All integrons share a common structure composed of a stable platform and a variable cassette array. The stable platform contains the following: (i) a gene encoding the integron integrase (*intI*), a site-specific tyrosine recombinase which catalyzes cassette rearrangements; (ii) a primary recombination site for the insertion of cassettes, *attI*; and (iii) a promoter, Pc, driving the expression of proximal cassettes in the array (Fig. 1A). The cassettes in the variable cassette array generally consist of a promoterless gene (coding sequence [CDS]) and a cassette recombination site (*attC*). Cassette arrays represent a low-cost repository of valuable functions for the cell and most likely reflect a history of adaptive events. The number of cassettes in the array can be very large in SCIs (more than 200), while it rarely exceeds eight in MIs (9, 10). Interestingly, *attC* sites found in SCI cassette arrays generally show a high degree of sequence identity, which increases with the number of cassettes (10). In contrast, *attC* sites of MI cassette arrays differ in length and sequence (11).

Integrons are atypical site-specific recombination systems. Unlike the *attI* sites, which are recombined in the classical double-stranded (ds) form, *attC* sites are recombined in a single-stranded (ss) folded form (Fig. 1B) (12–15). More precisely, the bottom strand of the *attC* site recombines about 10^3^ times more frequently than the top strand (14). The preference for the bottom strand ensures that cassettes are inserted in the correct orientation relative to the Pc promoter, allowing their expression (16, 17). In contrast to canonical site-specific recombination sites, the recognition of *attC* sites does not rely on the nature of their primary sequence but rather on the structure of their folded single-stranded DNA (ssDNA), as they share only 3 conserved nucleotides at the cleavage site (18). Folded *attC* sites form imperfect hairpins containing three unpaired structural features, the extrahelical bases (EHBs), the unpaired central spacer (UCS), and the variable terminal structure (VTS), which ensure strand selectivity and high levels of *attC* recombination (16–18). The variability of *attC* site length (from 57 to 141 bp) is mostly due to differences in the VTS loop (Fig. 1B) (19), which ranges from three unpaired nucleotides as in the *attC* site of the *aadA7* gene (*attC*_*aadA7*_ site) to complex branched secondary structures in larger sites such as *Vibrio cholerae* repeat (VCR) sites (the *attC* sites from *V. cholerae* SCI [20]). Due to the ss nature of the *attC* site, the *attI × attC* recombination generates, after the first strand exchange, an atypical and asymmetric Holliday junction. To complete the recombination event, this Holliday junction has to be resolved through replication (21).

In terms of cassette dynamics, recombination between *attC* sites leads to the excision of a cassette from the array. Recombination between the *attC* site of the excised cassette and the *attI* site allows for the reinsertion of the cassette in the beginning of the array, placing it downstream of the Pc promoter where it is more highly expressed. Hence, cycles of excision and insertion shuffle cassettes in the array and change their expression patterns (Fig. 1A) (22, 23). In addition, excision and loss of cassettes close to Pc may increase the expression of downstream cassettes. Such events observed in clinical settings (24) are probably more cost-effective than excisions followed by insertions (25). Stress responses, especially the SOS response, increase the expression of the integrase and accelerate the dynamics of integron shuffling and dissemination. This ensures a rapid and “on demand” adaptation to novel environmental contexts and limits pleiotropic effects in the host bacterium (26, 27). The entrance of ssDNA into the cell by conjugation or transformation can also induce the SOS response, thus coupling integrase expression to moments when incoming DNA could supply novel cassettes (23, 28).

Overall cassette dynamics depend on both cassette excision and insertion. The balance between the two processes determines whether the array accumulates or loses cassettes over time. Since cassette excision is a prerequisite step for further cassette insertion and as its regulation influences the rate of both processes, we focused our studies on excision dynamics. The rate of cassette excision must be a result of a trade-off between evolvability and genetic capacitance. The rate needs to be high enough to ensure shuffling and dissemination of cassettes (and the adaptive functions they encode). However, if the rate is too high and the balance between cassette excision and insertion is shifted toward excision, then cassettes could be rapidly lost, decreasing the probability of their vertical transmission. Since cassette excision directly depends on simultaneous folding of consecutive *attC* sites, the regulation of cassette excision is dependent on *attC* site folding, meaning that bacteria must regulate it subtly. This is particularly important because the presence of stable and long hairpin structures can also be detrimental for the maintenance of bacterial genomes (29). We have previously demonstrated that there is a subtle equilibrium between opposite processes: on one hand, *attC* site integrity ensured by the single-stranded DNA binding (SSB) protein which hampers folding of *attC* sites in the absence of the integrase (30); on the other hand, *attC* site folding and recombination favored by the availability of ssDNA (for instance, during conjugation and replication) and by the propensity to form cruciform structures due to supercoiling (31).

In order to gain a better understanding of cassette excision dynamics in integrons, we performed both *in silico* analyses and *in vivo* experiments to study the parameters that play important roles in maintaining an adequate level of cassette excision: orientation of cassette arrays relative to replication, CDS lengths, and *attC* site properties. Finally, we discuss the results obtained for MIs and SCIs in terms of integron evolutionary biology.

## RESULTS

### *In silico* analyses of integrons. (i) Orientation of cassettes relative to replication.

**FIG 2:**
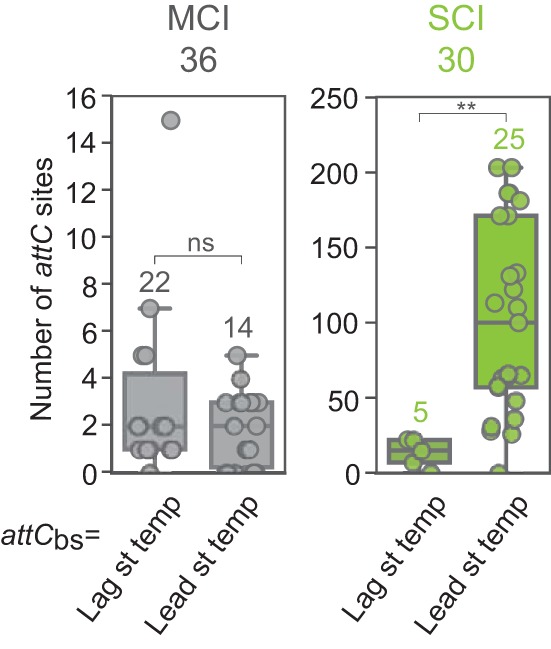
Distribution of the number of *attC* sites per integron (mobile chromosomal integron [MCI] and sedentary chromosomal integron [SCI]) as a function of *attC*_bs_ orientation relative to replication. Circles correspond to the number of *attC* sites for each of the analyzed 36 MCIs and 30 SCIs. Tests of the differences between data sets were performed using the Wilcoxon rank sum test (**, *P* value of 1.25 × 10^−3^; ns, not significant). bs, bottom strand; Lag st temp, lagging strand template; Lead st temp, leading strand template.

The differences in ssDNA availability between the lagging and leading strands during DNA replication affect the formation of DNA secondary structures (32). When the bottom strand of an *attC* site (*attC*_bs_) is located on the lagging strand template in which large regions of ssDNA are available (i.e., between Okazaki fragments), its folding is favored, increasing the frequency of *attC* × *attI* recombination (31). We previously observed that in all 10 analyzed sedentary chromosomally located integrons (SCIs), *attC* sites were oriented so that their bottom strands were located on the leading strand template, potentially limiting cassette rearrangements (31). Here, we broadened this analysis by comparing 30 SCIs with 36 mobile chromosomal integrons (MCIs) (Fig. 2; see Data Set S1 in the supplemental material). The latter corresponded to mobile integrons (carried on transposons) but located in chromosomes. We confirmed the previously observed trend: most SCIs (25/30) were oriented so that their *attC*_bs_ were located on the leading strand template. In particular, this was always the case for *Vibrio* species. We did not observe such bias in orientation among MCIs.

10.1128/mBio.02296-16.10 DATA SET S1  Integrons identified by IntegronFinder. Download DATA SET S1, XLSX file, 0.1 MB. Copyright © 2017 Loot et al. 2017 Loot et al. This content is distributed under the terms of the Creative Commons Attribution 4.0 International license.

We hypothesized that the orientation of the integron array relative to replication affects cassette dynamics by modifying their excision rate. Indeed, SCIs with *attC*_bs_ on the leading strand template had larger arrays of cassettes than the others (Fig. 2). More precisely, they carried up to 203 cassettes (median of 100), while arrays with *attC*_bs_ on the lagging strand template were always smaller (up to 22 cassettes, with a median of 15). In the data set of SCI-containing genomes, *Vibrio* genomes are overrepresented, reflecting the general bias toward human pathogen species. We therefore repeated this analysis after exclusion of *Vibrio* genomes and found that this difference remained significant (Fig. S1).

10.1128/mBio.02296-16.1 FIG S1  Distribution of the number of *attC* sites per integron (MCI and SCI) as a function of *attC*_bs_ orientation relative to replication excluding *Vibrio* strains. Dots correspond to the number of *attC* sites for each of the analyzed 34 MCIs and 9 SCIs. Tests of the differences between data sets were performed using the Wilcoxon rank sum test (*, *P* value of 1.43 × 10^−2^; ns, not significant). MCI, mobile chromosomal integron; SCI, sedentary chromosomal integron; bs, bottom strand; Lag st temp, lagging strand template; Lead st temp, leading strand template. Download FIG S1, EPS file, 0.5 MB. Copyright © 2017 Loot et al. 2017 Loot et al. This content is distributed under the terms of the Creative Commons Attribution 4.0 International license.

### (ii) Cassette lengths.

**FIG 3:**
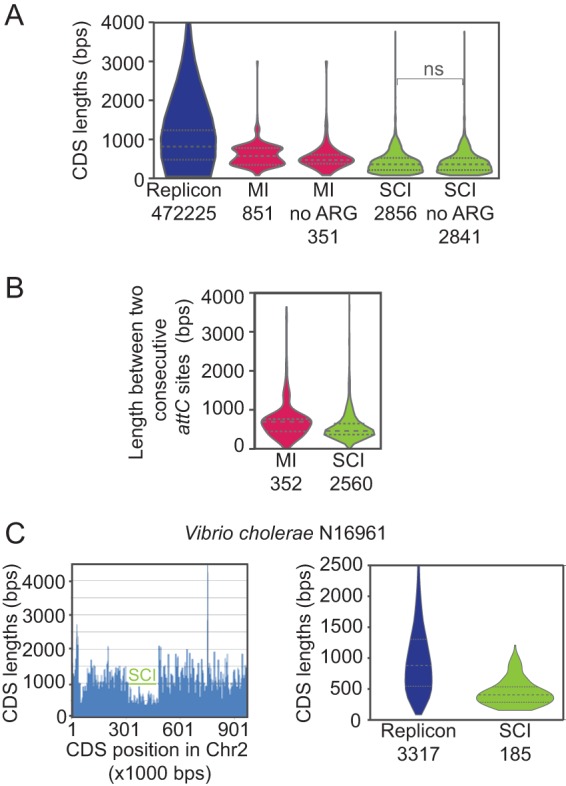
Cassette and coding sequence (CDS) length analysis. The length of the CDS in replicons, mobile integrons (MI), and sedentary chromosomal integrons (SCI) with and without antibiotic resistance genes (ARG) is shown in base pairs (bps). The numbers below each violin diagram refer to the total number of CDSs or cassettes analyzed. (A) Violin plots showing the distribution of CDS lengths for replicons, MIs, and SCIs (excluding ARGs or not excluding ARGs). Tests of the differences between data sets were performed using the Wilcoxon rank sum test. Differences are significant (*P* values of <10^−6^), except between the two rightmost violin plots (differences between CDS lengths of all SCIs and SCIs excluding ARGs or not excluding ARG). ns, not significant. (B) Violin plots showing the distribution of lengths between two consecutive *attC* sites for MIs and SCIs. Tests of the differences between data sets were performed using the Wilcoxon rank sum test. Differences are significant (*P* values of <10^−6^). (C) CDS length analysis of the *Vibrio cholerae* N16961 strain. **(**Left) CDS lengths as a function of their positions in chromosome 2 (Chr2). The horizontal green bar indicates the position of the SCI. (Right) Violin plots showing the distribution of CDS lengths for the two replicons and the SCI. Tests of the differences between data sets were performed using the Wilcoxon rank sum test. Differences are significant (*P* values of <10^−6^).

Cassette lengths were investigated as another means of control over their excision dynamics. When simultaneous folding of both flanking *attC* sites is promoted, e.g., when the distance between both sites was smaller than the length of ssDNA found between two Okazaki fragments or smaller than the size of supercoiled plectonemes, cassette excision is likely to be favored. Therefore, we decided to analyze the cassette length. We used two independent proxies for the length of cassettes to test this hypothesis: CDS length and the distance between identified *attC* sites. On the one hand, CDS length underestimates cassette length but is highly correlated with it, since most cassettes have one single CDS and small adjacent regions. On the other hand, the distance between *attC* sites provides an exact measure of cassette length but is affected by inaccuracies in the detection of *attC* sites (a missed *attC* can lead to the doubling of a cassette length). We performed these analyses for the 393 integrons identified by the IntegronFinder program (10) (Materials and Methods) and their respective replicons. CDSs in MIs and SCIs are significantly shorter than CDSs in replicons (median CDS lengths were 575, 362, and 818 bp, respectively [Fig. 3A]). Moreover, CDSs in SCIs are significantly shorter than those in MIs. We also performed this analysis by excluding known antibiotic resistance genes (ARGs) because they are overrepresented in MIs (500 out of 851 CDSs), upon which we observed a significant decrease in median MI CDS lengths. However, the non-ARG CDSs in MIs remain significantly longer than the non-ARG CDSs in SCIs (median CDS lengths were 473 and 362 bp, respectively).

We also measured the lengths of cassettes using the distance between two consecutive *attC* sites (Fig. 3B), which confirmed that cassettes of SCIs are smaller than those of MIs. We made three controls to validate these results. First, we excluded *Vibrio* genomes because they are overrepresented (Fig. S2A). Second, we controlled for interintegron and interreplicon variability by determining the mean values of CDS lengths per integron or per replicon and the mean lengths between two consecutive *attC* sites per integron (Fig. S2B and S2C). Third, we calculated pairwise differences in mean CDS lengths between a replicon and its associated integron to control for interspecies variability (Fig. S2D).

10.1128/mBio.02296-16.2 FIG S2  Cassette and CDS length analysis of the 393 integron-containing strains identified by IntegronFinder and their replicons. Tests of the differences between data sets were performed using the Wilcoxon rank sum test. Differences are significant (*P* values of <0.05) except in Fig. S2A and S2B between the two rightmost violin plots. bps, base pairs; MI, mobile integron; SCI, sedentary chromosomal integron; ns, not significant; ARG, antibiotic resistance genes. The numbers below each violin diagram refer to the total number of CDS, replicons, or integrons analyzed. (A) Violin plots showing the distribution of CDS lengths for replicons, MIs, and SCIs (excluding ARGs or not excluding ARGs) excluding *Vibrio* strains. (B) Violin plots showing the distribution of mean CDS lengths per replicon and per integron for replicons, MIs, and SCIs (excluding ARGs or not excluding ARGs). (C) Violin plots showing the distribution of mean lengths between two consecutive *attC* sites per integron for MIs and SCIs. (D) Violin plots showing the distribution of pairwise differences in mean CDS lengths between a replicon and its integron for MIs and SCIs. Download FIG S2, EPS file, 1 MB. Copyright © 2017 Loot et al. 2017 Loot et al. This content is distributed under the terms of the Creative Commons Attribution 4.0 International license.

A clear example of this difference in lengths between CDSs of SCIs and replicons is the paradigmatic SCI of *V. cholerae*. CDSs in this SCI are significantly shorter than CDSs in the replicon (median CDS lengths are 405 and 882 bp, respectively [Fig. 3C]). We extended our analysis to SCIs present in other *Vibrio* species and observed similar trends (Fig. S3).

10.1128/mBio.02296-16.3 FIG S3  CDS length analysis of *Vibrio* strains and their SCIs. The horizontal green bar indicates the position of the SCI. bps, base pairs; CDS, coding sequence. (A) CDS lengths as a function of their positions in chromosome 1 (Chr1) of *Vibrio vulnificus* CMCP6 strain. (B) CDS lengths as a function of their positions in chromosome 2 (Chr2) of *Vibrio fischerii* MJ11 strain. (C) CDS lengths as a function of their positions in chromosome 1 (Chr1) of *Vibrio parahaemolyticus* RIMD 2210633 strain. (D) CDS lengths as a function of their positions in chromosome 2 (Chr2) of *Aliivibrio salmonicida* LFI1238 strain. (E) CDS lengths as a function of their positions in chromosome 1 (Chr1) of *Vibrio alginolyticus* ATCC 17749 strain. (F) CDS lengths as a function of their positions in the whole genome of *Vibrio rotiferanus* DAT722 strain. Download FIG S3, PDF file, 0.6 MB. Copyright © 2017 Loot et al. 2017 Loot et al. This content is distributed under the terms of the Creative Commons Attribution 4.0 International license.

### (iii) *attC* site properties.

**FIG 4:**
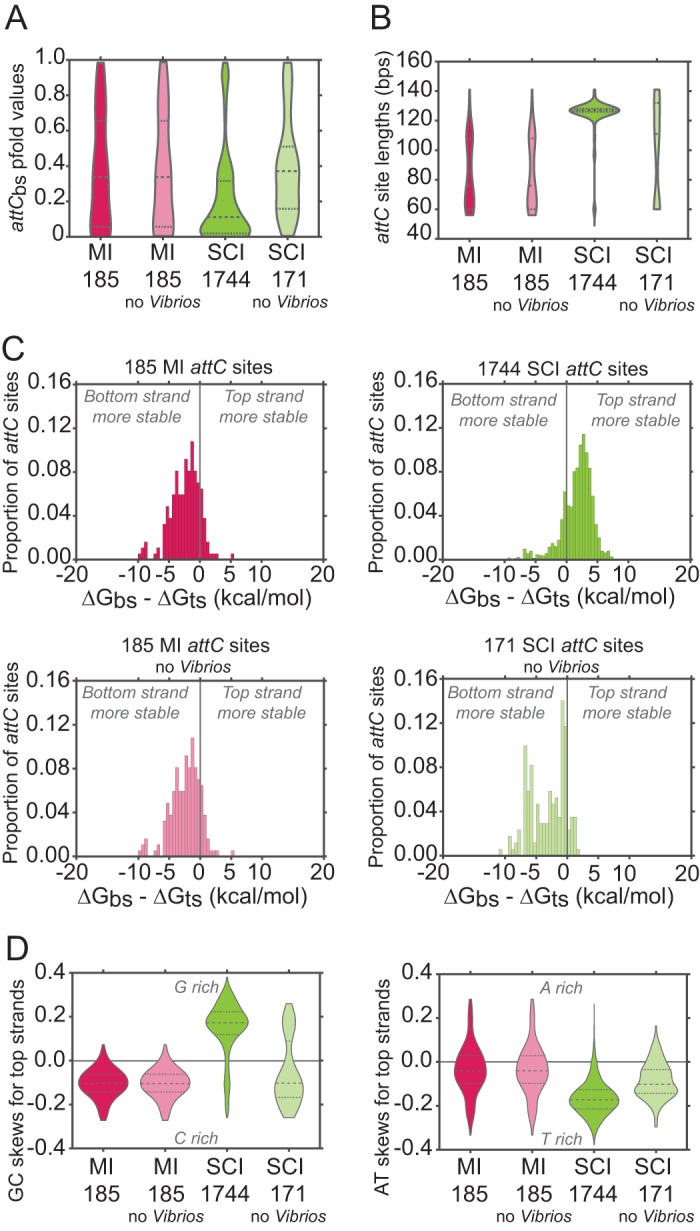
Analysis of 185 mobile integron (MI) and 1,744 sedentary chromosomal integron (SCI) *attC* site properties. *attC* sites were identified by IntegronFinder (Materials and Methods). The numbers below each violin diagram refer to the total number of *attC* sites analyzed. bs, bottom strand; ts, top strand; bps, base pairs. (A) Violin plots showing the distribution of *attC*_bs_ pfold values for MIs and SCIs, excluding *Vibrio* strains or not excluding *Vibrio* strains. *attC*_bs_ pfold values were calculated with RNAfold program from the ViennaRNA 2 package (Materials and Methods). Tests of the differences between data sets were performed using the Wilcoxon rank sum test. Differences are significant (*P* values of <10^−6^), except between MI *attC* sites excluding *Vibrio* strains or not excluding *Vibrio* strains, and between SCI *attC* sites excluding *Vibrio* strains and MI *attC* sites (excluding *Vibrio* strains or not excluding *Vibrio* strains). (B) Violin plots showing the distribution of *attC* site lengths for MIs and SCIs, excluding *Vibrio* strains or not excluding *Vibrio* strains. Tests of the differences between data sets were performed using the Wilcoxon rank sum test. Differences are significant (*P* values of <10^−4^) except between MI *attC* sites excluding *Vibrio* strains or not excluding *Vibrio* strains. (C) Proportion of *attC* sites as a function of the difference in Δ*G* between bottom and top strands (ΔG_bs_ − ΔG_ts_) for MIs and SCIs, excluding *Vibrio* strains or not excluding *Vibrio* strains. Δ*G* values (in kilocalories per mole) were calculated with RNAfold program from the ViennaRNA 2 package (Materials and Methods). (D) Violin plots showing the distribution of GC and AT skews calculated for the top strands of the *attC* sites for MIs and SCIs, excluding *Vibrio* strains or not excluding *Vibrio* strains. GC and AT skews were calculated as described in Materials and Methods. Negative skews correspond to an enrichment of purines (guanines [G] or adenines [A]) on the bottom strands. C, cytosine; T, thymine.

Cassette insertion frequency depends on the properties of *attC* sites involved in the reaction (17, 31). In order to be bound by the integrase, *attC* sites must adopt a recombinogenic structure, i.e., with paired R and L boxes (Fig. 1B). Based on DNA folding predictions, the probability of folding a recombinogenic structure can be calculated, which we call the pfold value (Materials and Methods). The presence of a large VTS can favor the formation of complex branched structures that do not reconstitute a recombinogenic *attC* site (31). Therefore, the length of *attC* sites could be an important parameter influencing their recombination. We compared the properties of *attC* sites in MIs and SCIs that are most likely to affect recombination levels: pfold, length of *attC* sites, and stability of the recombinogenic structure once folded (Δ*G*). These analyses were performed on 185 *attC* sites from MIs and 1,744 *attC* sites from SCIs (Fig. 4) (Materials and Methods).

We observed significant differences between MI and SCI *attC* site pfold values (Fig. 4A and S4A). The large majority of MI *attC* sites have a very high pfold value (71% sites with a pfold value of >0.1), whereas SCIs contain fewer such sites (only 52%). Moreover, contrary to MIs, SCIs contain sites with an extremely low pfold value (6% sites with a pfold value between 10^−5^ and 10^−7^). These low-pfold *attC* sites are mostly found in *Vibrio* SCIs. When *attC* sites exclusively found in *Vibrio* strains were excluded from the data set, we found no significant difference among the pfold values of MI and SCI sites (Fig. 4A and S4A).

10.1128/mBio.02296-16.4 FIG S4  Supplementary data on *attC* site lengths and pfold values. MI, mobile integron; SCI, sedentary chromosomal integron. (A) Proportion of *attC* sites as a function of *attC*_bs_ pfold values for MIs and SCIs, excluding *Vibrio* strains or not excluding *Vibrio* strains. bs, bottom strand. (B) Proportion of *attC* sites as a function of the length of the *attC* site for MIs and SCIs, excluding *Vibrio* strains or not excluding *Vibrio* strains. bps, base pairs. Download FIG S4, EPS file, 1.3 MB. Copyright © 2017 Loot et al. 2017 Loot et al. This content is distributed under the terms of the Creative Commons Attribution 4.0 International license.

*attC* site length comparison between MIs and SCIs showed that the former have smaller VTSs, which can be as short as 3 nucleotides. The *attC* sites of SCIs often have longer VTSs. The length of MI *attC* sites is relatively heterogeneous, ranging from 56 to 141 bp, with a majority of small *attC* sites (<100 bp) (Fig. 4B and S4B). The *attC* sites of SCIs are more homogeneous, predominantly measuring between 120 and 129 bp (Fig. 4B and S4B). However, this size distribution is mostly due to *attC* sites of *Vibrio* spp. When the *Vibrio attC* sites are excluded, the length of *attC* sites is not significantly different between MIs and SCIs (Fig. 4B and S4B). We did not observe any correlation between the length and the pfold values of *attC* sites, even when excluding the *Vibrio attC* sites (Fig. S5A).

10.1128/mBio.02296-16.5 FIG S5  Supplementary data on *attC* site properties. For each data set, the slope of the regression, as well as the corresponding coefficient of determination *R*^2^ and *P* value are indicated. MI, mobile integron; SCI, sedentary chromosomal integron; bps, base pairs; bs, bottom strand. (A) *attC*_*bs*_ pfold values as a function of *attC* site lengths for MIs and SCIs, excluding *Vibrio* strains or not excluding *Vibrio* strains. (B) Difference in Δ*G* between the bottom and top strands (Δ*G*_bs_ − Δ*G*_ts_) of *attC* sites as a function of their length for MIs and SCIs, excluding *Vibrio* strains or not excluding *Vibrio* strains. Download FIG S5, JPG file, 1 MB. Copyright © 2017 Loot et al. 2017 Loot et al. This content is distributed under the terms of the Creative Commons Attribution 4.0 International license.

Our previous study of 263 MI *attC* sites from the INTEGRALL database (33) showed that the Δ*G* of the folded bottom strands (Δ*G*_bs_) is on average 2.12 kcal/mol lower than the Δ*G* of the folded top strands (Δ*G*_ts_), suggesting that folded bottom strands are more stable (17). The 185 MI *attC* sites from our genomic data set show similar differences (2.38 kcal/mol [Fig. 4C]). Surprisingly, the analysis of 1,744 SCI *attC* sites shows that Δ*G*_bs_ is on average 1.71 kcal/mol higher than the Δ*G*_ts_, suggesting that folded top strands are more stable. Once again, this effect was due to the *attC* sites of *Vibrio* spp.: their exclusion reversed the trend toward higher Δ*G*_ts_ (differences of 3.13 kcal/mol). This value was not significantly different from the one observed for MI *attC* sites (Fig. 4C). We observed a negative correlation between the length of *attC* sites and the Δ*G*_bs_ − Δ*G*_ts_ in MIs and in SCIs without *Vibrio attC* sites (Fig. S5B). This correlation was reversed for SCI *attC* sites, which is expected, given that their increased length is mostly due to a longer VTS, which together with the UCS produces this difference in Δ*G* (Fig. S5B).

As previously described, the bottom strands of MI *attC* sites are enriched in purines, especially in guanines, which contribute to the difference in folded strand stability (17). Indeed, purines have a higher self-stacking tendency, thus stabilizing secondary structures (34). We confirmed negative GC and AT skews for MI *attC* sites from our data set (the skews were calculated relative to the top strands) (Fig. 4D). However, for SCI *attC* sites, we observed positive GC and negative AT skews, meaning that the bottom strands were C and A rich. This difference in nucleotide skews of *attC* sites in MIs and SCIs could explain, at least in part, the difference in folded strand stability between bottom and top strands. When *attC* sites from *Vibrio* spp. were excluded from the analysis, the GC skew of the remaining SCI *attC* sites became negative as in MI sites, even though there was a small subpopulation of SCI *attC* sites with C-rich bottom strands (Fig. 4D). Additionally, these remaining SCI *attC* sites showed a more homogenous negative AT skew, resembling that of MI sites.

### *In vivo* analysis of cassette excision. (i) Cassette excision assay.

**FIG 5:**
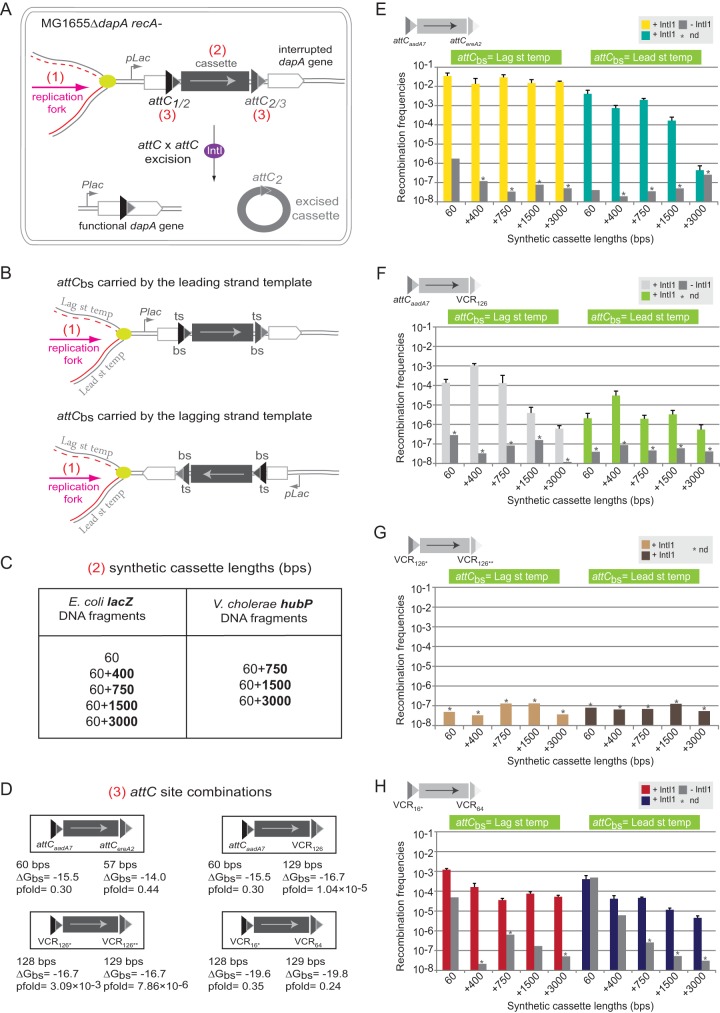
Cassette excision assay. (A) Experimental set-up of the cassette excision assay. The *dapA* gene (white rectangle) is interrupted by a synthetic cassette containing a DNA fragment (gray rectangle) flanked by two *attC* sites (triangles). Recombination mediated by the IntI integrase (purple oval) leads to the excision of the cassette (excised cassette [gray circle]) and restores a functional *dapA* gene. Three parameters are varied: (1) orientation of cassettes relative to replication; (2) cassette lengths; and (3) different *attC* site combinations. (B) Orientation of cassettes relative to replication. The orientation of the bottom strands (bs) of *attC* sites relative to replication are shown. Lag st temp, lagging strand template; Lead st temp, leading strand template. (C) Synthetic cassette lengths. Lengths in base pairs (bps) of the supplementary *E*. *coli lacZ* and *V. cholerae hubP* DNA fragments introduced in synthetic cassettes are indicated in bold numbers. (D) *attC* site combinations. The four *attC* site combinations used are shown. Δ*G*_bs_ (in kilocalories per mole) and *attC*_bs_ pfold were calculated with RNAfold program from the ViennaRNA 2 package (Materials and Methods, and Fig. S6). Δ*G*_bs_ is calculated for the most stable structure with constraints (recombinogenic) (Fig. S6). The numbering of each VCR indicates its position in the *V. cholerae* N16961 SCI. The single and double asterisks indicate that the wild-type *attC* site has been slightly modified (by removing 1 nucleotide [nt] [single asterisk] or changing 1 nt [double asterisk]) in order to generate a functional *dapA* fusion after recombination events. bs, bottom strand. (E to H) Recombination frequencies for *attC*_*aadA7*_-*attC*_*ereA2*_ (E), *attC*_*aadA7*_-VCR_126 _(F), VCR_126*-_VCR_126**_ (G), and VCR_16*-_VCR_64 _(H) synthetic cassettes. Synthetic cassette lengths and orientation of *attC*_*bs*_ relative to replication are indicated. Experiments were performed in the presence (+) or absence (−) of IntI1 integrase. bs, bottom strand; Lag st temp, lagging strand template; Lead st temp, leading strand template; bps, base pairs; nd, not detected.

In order to better understand the biological significance of our *in silico* analyses, we performed *in vivo* excision tests for several synthetic cassettes using the previously described excision assay (23) (Fig. 5A). In this assay, excision of cassettes between *attC* sites leads to reconstitution of the essential *dapA* gene, allowing recombinants to grow on media lacking 2,6-diaminopimelic acid (DAP) (the reticulating agent of peptidoglycan in *Escherichia coli*). Comparison of the number of clones growing with and without DAP yields a recombination frequency for a given reaction. Corresponding strains without integrase were used as controls to assess the rate of false-positive events potentially due to replication slippage.

We tested the influence of three parameters on cassette excision (Fig. 5A): (i) orientation of cassettes relative to replication, (ii) cassette lengths, and (iii) different *attC* site combinations. To study the effect of cassette orientation, we inserted synthetic cassettes into the λ *attB* site of the MG1655*ΔdapA* strain in both orientations, so that the bottom strands of both *attC* sites were on either the leading or lagging strand template (Fig. 5B). For the leading strand template orientation, the only possibility for *attC* sites to fold was by extrusion from dsDNA. For the lagging strand template orientation, *attC* sites could fold either by extrusion from dsDNA or directly from ssDNA generated from discontinuous replication. We varied the cassette length by introducing DNA fragments of various lengths (from 60 to 3,060 bp) between two *attC* sites (*E. coli lacZ* DNA fragments) (Fig. 5C). Finally, we also used four combinations of *attC* sites: *attC*_*aadA7*_-*attC*_*ereA2*_, *attC*_*aadA7*_-VCR_126_, VCR_126*_-VCR_126**_, and VCR_16*_-VCR_64_ (VCR names explained in the legend to Fig. 5D) (Fig. 5D and S6). While *attC*_*aadA7*_ and *attC*_*ereA2*_ are found in MIs, the VCRs correspond to *attC* sites from the *V. cholerae* SCI.

10.1128/mBio.02296-16.6 FIG S6  Supplementary data on *attC* sites used in synthetic cassettes. Download FIG S6, PDF file, 0.2 MB. Copyright © 2017 Loot et al. 2017 Loot et al. This content is distributed under the terms of the Creative Commons Attribution 4.0 International license.

We performed two additional controls on *attC*_*aadA7*_-*attC*_*ereA2*_ and *attC*_*aadA7*_-VCR_126_ cassettes. First, we tested that the expression of *dapA* from the P_*lac*_ promoter (Fig. 5A) did not interfere with recombination by performing excision reactions without isopropyl-β-d-thiogalactopyranoside (IPTG). Second, we tested for sequence-specific effects by replacing selected *lacZ* DNA fragments by *hubP* fragments from *V. cholerae* (Fig. 5C). In all these controls, we found no significant difference from our results (Fig. S7).

10.1128/mBio.02296-16.7 FIG S7  Recombination frequencies for different synthetic cassettes in control experiments. Cassette excision assay was performed for several synthetic cassette combinations: *attC*_*aadA7*_-*attC*_*ereA2*_ (A and C) and *attC*_*aadA7*_-VCR_126_ (B and D). Experiments were performed in the presence (+) or absence (−) of the IntI1 integrase. Synthetic cassette lengths and orientation of *attC*_bs_ relative to the replication are indicated. (A and B) Experiments were performed in the presence (+) and absence (−) of IPTG. (C and D) *V. cholerae hubP* DNA fragments were used instead of *E. coli lacZ* DNA fragments. bs, bottom strand; Lag st temp and Lead st temp, lagging and leading strand template, respectively; bps, base pairs; nd, not detected events. Download FIG S7, EPS file, 1.1 MB. Copyright © 2017 Loot et al. 2017 Loot et al. This content is distributed under the terms of the Creative Commons Attribution 4.0 International license.

### (ii) Effects of regulatory network on cassette excision.

*(a)* attC_aadA7_-attC_ereA2_ *synthetic cassettes*. The first set of synthetic cassettes was flanked by *attC*_*aadA7*_ and *attC*_*ereA2*_ MI sites (Fig. 5D). The pfold value of both sites is >0.1, implying that their most stable structures are recombinogenic (Fig. 5D and S6). Recombination occurred at high frequency for all cassettes when *attC*_bs_ were carried on the lagging strand template and for all but the largest cassette when *attC*_bs_ were carried on the leading strand template (Fig. 5E). This suggests that both tested *attC* sites could be efficiently and simultaneously extruded from dsDNA (Fig. 5E). In the absence of integrase, we observed excision events only for small cassettes (60-bp-long cassette) in both orientations, probably due to replication slippage (Fig. S8) (30).

10.1128/mBio.02296-16.8 FIG S8  False-positive recombination events due to slippage. Direct repeats of *attC*_*aadA7*_ (gray) and *attC*_*ereA2*_ (orange) are shown in red. (A) Sequences of *attC*_*aadA7*_, *attC*_*ereA2*_, and the slippage-resulting *attC* site. The sequences of the top strands are shown. (B) Proposed model for the slippage events. The model proposed is that *attC* site folding permits a slipped mispairing mechanism during replication, resulting in a cassette excision event, which reconstitutes a functional *dapA* gene independently of integrase expression. This scheme represents the *attC*_bs_ carried on the leading strand template, but in cases where the *attC*_bs_ is carried on the lagging strand template, the slippage mechanism is the same. bs, bottom strand; ts, top strand; Lag st temp, lagging strand template; Lead st temp, leading strand template. Download FIG S8, EPS file, 1 MB. Copyright © 2017 Loot et al. 2017 Loot et al. This content is distributed under the terms of the Creative Commons Attribution 4.0 International license.

*(b)* attC_aadA7_-*VCR*_*126*_ *synthetic cassettes.* In order to assess the importance of the *attC* site pfold value on cassette excision, we tested the excision of cassettes flanked on one side by the previously used *attC*_*aadA7*_ site and on the other side by the VCR_126_ site. VCR_126_ has a very low pfold value (pfold value of 1.04 × 10^−5^) that is significantly lower than that of *attC*_*ereA2*_ (Fig. 5D and S6), and it is also unlikely to fold into a recombinogenic structure from dsDNA (31). Accordingly, we observed significantly lower recombination rates for this cassette independently of cassette orientation and fragment type (Fig. 5F and S7D). As for the previous set of cassettes, when *attC*_bs_ were carried on the lagging strand template, the frequency of recombination was higher than in the inverse orientation. However, for this set of cassettes, recombination also depended on cassette length (Fig. 5F). Due to the low propensity of the VCR site to extrude from dsDNA, we observed a relatively constant low frequency of cassette excision when the bottom strands of *attC* sites were carried on the leading strand template.

*(c) VCR-VCR synthetic cassettes*. We also tested two sets of cassettes flanked on both sides by VCR sites. First, we combined VCR_126*_ and VCR_126**_ sites. The most stable structures of these sites are non-recombinogenic (pfold values of 3.09 × 10^−3^ and 7.86 × 10^−6 ^[Fig. 5D and S6]), and we expected that the low pfold values would not allow simultaneous folding and cassette excision from the ds pathway or even the ss pathway. Indeed, we did not detect excision of these cassettes (Fig. 5G). Second, we combined VCR_16*_ and VCR_64_ sites for which the most stable structures are recombinogenic (pfold values of 0.35 and 0.24 [Fig. 5D and S6]). In this case, we observed high rates of recombination from the ds and/or ss pathway (Fig. 5H), presumably because both VCR sites could fold efficiently. We observed higher recombination rates when *attC*_bs_ were carried on the lagging strand template. The frequency of recombination, similarly to *attC*_*aadA7*_-VCR_126_ cassettes, depended on cassette length when *attC*_bs_ were carried on the lagging strand template (from 1.17 × 10^−3^ for 60 bp to 5.25 × 10^−5^ for +3,000-bp cassette lengths), but this effect was less pronounced for VCR_16*_-VCR_64_ cassettes (Fig. 5F and H). As for other sites, in the absence of integrase, we observed recombination events only for small cassettes (60- and +400-bp cassette lengths) in both orientations. These events were likely due to replication slippage favored by the high sequence identity between the two sites (83% [Fig. S8]).

## DISCUSSION

Our study aimed to understand the rules that govern cassette array dynamics in SCIs and MIs and to determine the cause of shorter CDS lengths in cassettes compared to the rest of the genome.

### Replication controls cassette dynamics in integrons.

The *in silico* analyses reveal that *attC*_bs_ in SCIs are predominantly carried on the leading strand template and that these SCI arrays are significantly larger, up to 200 cassettes in SCIs of *Vibrio* spp. Our *in vivo* results show that such orientation relative to replication limits cassette excision and thus stabilizes large cassette arrays. This may explain why this orientation was much less frequent among MCIs, for which a higher cassette mobility might be favored over the preservation of a larger reservoir of genetic functions that will stay accessible through horizontal gene transfer. Interestingly, the five SCIs identified in inverse orientation were found exclusively in *Xanthomonas* species and their number of cassettes did not exceed 22. Comparison of SCIs in these two closely related genera is particularly interesting, since according to phylogenetic analyses, the acquisition of integron systems in both *Vibrio* and *Xanthomonas* occurred independently and thus can be regarded as two single ancestral events (3, 35). Contrary to the *Vibrio* SCIs, the *Xanthomonas* SCIs are subjected to genetic erosion, and it is tempting to speculate that this is a consequence of their orientation and that the frequent inactivation of their integrases was selected to freeze cassette excisions (36).

### Cassette length constraints in integrons.

Our analyses revealed that CDSs carried on SCI and MI cassettes were on average shorter than the remaining replicon’s CDSs. In order to clarify the origin(s) of this characteristic, in particular whether the reduced length of CDSs in cassettes reflected a constraint in maximal distance between two consecutive *attC* sites for efficient recombination, we tested the effect of cassette length on their excision *in vivo*. We would then expect to see a significant drop in recombination frequency for cassettes larger than most SCI and MI cassettes and smaller than most replicon CDSs (that is, between 500 and 800 bp), at least in one orientation. However, this was not the case (Fig. 5). When bottom strands were carried on the lagging strand template, even though cassette length can have an impact on cassette excision frequency, we observed a consistent drop in recombination frequencies only for very large cassettes (≥1,500 bp), when at least one of the adjacent sites has a low pfold value (Fig. 5F). Also, we did not observe any consistent drop in recombination frequencies for cassettes up to 1,500 bp when *attC*_bs_ were carried on the leading strand template (Fig. 5). Taken together, these observations indicate that the reduced length of CDSs in cassettes is not due to a recombination-related limitation. It is possible that the prevalence of small CDSs in cassettes reflects constraints during cassette genesis. The process of *de novo* cassette formation is largely unknown, and the proposed hypotheses have many incongruities (discussed in reference 7). Thus, we can only speculate on the underlying processes and related constraints. However, the limitations in maximal cassette length are not stringent, since long cassettes such as ARGs encoding class D β-lactamases are found among MIs (37). Long cassettes might be less likely to be created, and their presence could reflect their strong selection and confer important selective advantages.

### *attC* site properties control cassette dynamics in integrons.

By using different *attC* sites in our synthetic cassettes, we tested the impact of their properties on cassette excision. When cassettes were flanked by two *attC* sites with high pfold values, cassette length did not influence the recombination rate when the bottom strands were carried on the lagging strand templates. Because of their high pfold values, simultaneous folding of the two *attC* sites could occur in three possible ways. (i) Both sites fold from ssDNA during the passage of the replication fork (the “ss pathway”). (ii) Both sites are extruded from dsDNA (the “ds pathway”). (iii) One site is folded from ssDNA, and the other is extruded from dsDNA. The third pathway could explain the very high efficiency of recombination that we obtained for +3,000-bp cassettes when bottom strands were carried on the lagging strand template. On the other hand, the difficulties of recombining +3,000-bp cassettes when both high-pfold *attC*_bs_ were carried on the leading strand template, and therefore must have been extruded from dsDNA, might be explained by topological constraints such as the presence of independent topological domains in bacterial chromosomes (38). Another hypothesis is that cruciform extrusion induces DNA structural transitions, restricting the slithering of the molecule and reducing the possibility of distant sites to come into contact (39).

When cassettes are flanked by at least one *attC* site with a low pfold value, we observed a decrease in recombination efficiency. Moreover, when cassettes are flanked on both sides by low-pfold *attC* sites, their excision frequency is even further decreased. In addition, the excision rate of cassettes flanked by high-pfold VCRs is decreased compared to that of cassettes flanked by high-pfold MI *attC* sites. These differences could be due either to the influence of other folding-related VCR properties or to host factor binding. Once folded, large VCR sites could be efficiently targeted by hairpin or cruciform-binding proteins (29).

Interestingly, we also observed an effect of cassette length on the excision frequency of cassettes flanked by at least one low-pfold *attC* site and oriented with the bottom strand carried on the lagging strand template. Under these conditions, cassette length correlates with cassette excision frequency, possibly because of a higher chance for two consecutive *attC*_bs_ sites to be located within the ss region at the replication fork (between Okazaki fragments [40]). This effect has previously been observed for the IS*608* insertion sequence, which also requires ssDNA substrates to recombine (41).

These results show that *attC* site pfolds, and more generally *attC* site biophysical properties, control cassette excision dynamics. Moreover, the *in silico* analyses demonstrated that MIs mostly contain small *attC* sites with high pfold values and folded bottom strands which are more stable than folded top strands. This ultimately favors their recombination by the integrase (17). This is also true for many SCIs, but surprisingly, not for *Vibrio* SCIs. The reason for such discrepancy is unknown, but the genomic architecture of vibrios, with their two-chromosome replication being highly regulated and coordinated and their unique physical organization (42, 43) might be at the origin of several specific traits.

### Cassette dynamics: evolutionary considerations and trade-off.

In these studies, we show that cassette excision is highly regulated by the cell replication process and the properties of cassette recombination sites. We demonstrate that differential dynamics of cassette excision are ensured by integron properties that shape a trade-off between evolvability and genetic capacitance. In MIs, efficient cassette recombination is favored and timed to conditions when generating diversity upon which selection can act ensures a rapid response to environmental stresses. In contrast, in SCIs, cassette dynamics favor the maintenance of large cassette arrays and vertical transmission. Interestingly, even in large SCI arrays, there are very few pseudogenes among cassette CDSs. Several studies of *V. cholerae* have shown that the SCI array was the most variable locus among isolates (44, 45). This suggests that on a global time scale, cassettes must be regularly rearranged and tested for selective advantage, thus explaining the preservation of cassette gene functionality, even when promoterless. We therefore confirmed the role of SCIs as a reservoir of adaptive functions. Indeed, the evolutionary history of integrons suggests that SCIs could constitute a cassette reservoir and that subsequent harvesting of cassettes from various SCI sources leads to contemporary MIs (35). SCI *attC* sites display a strikingly high degree of sequence relatedness (around 80% identity), unlike their MI counterparts (3, 10). This suggests a link between the host and the sequences of *attC* recombination sites, and more precisely, it suggests that the formation of *de novo* integron cassettes most likely occurs in SCI hosts. Moreover, SCI cassettes can become substrates of MI integrases and therefore be directly recruited into MIs as demonstrated for class 1 integrons (3, 20, 46). Thus, the most recombinogenic cassettes in SCIs would be more likely mobilized in MIs and further selected because of their higher capacity to disseminate the associated adaptive functions. The ensemble of these regulation processes can have a direct effect on integrase stability. A mathematical model has suggested that, while integrases in MIs are selectively maintained by the antibiotic pressure, integrases in SCIs are maintained because they enable their hosts to use cassette arrays efficiently as a reservoir of standing genetic variability (47).

**FIG 6:**
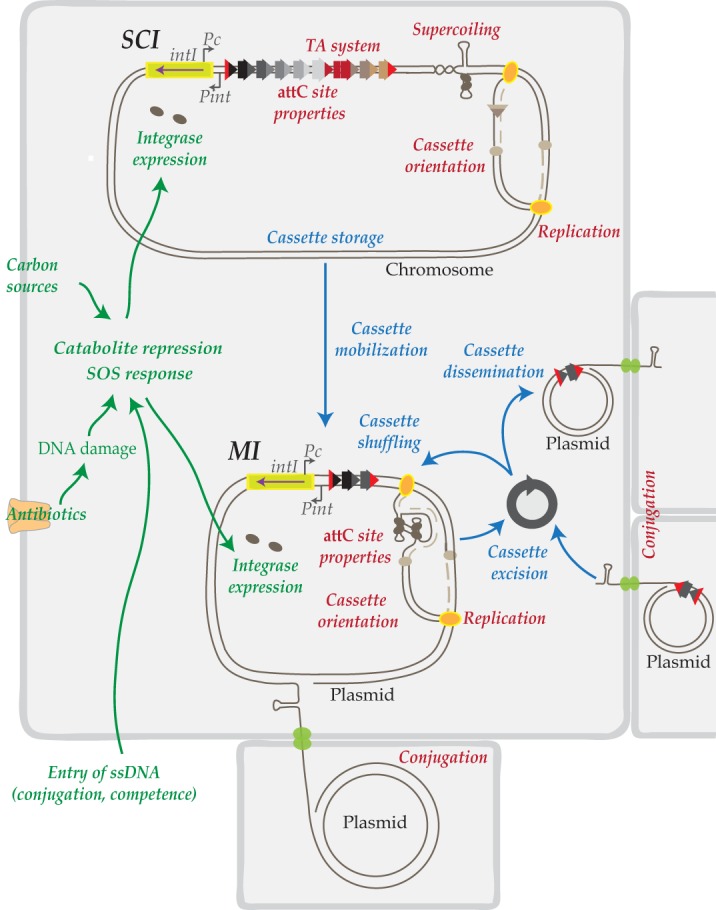
Regulatory network in integrons. Representation of cassette dynamics, namely, cassette storage in SCIs, cassette mobilization from SCIs to MIs, and cassette excision, shuffling, and dissemination in MIs is shown in blue. Representation of the regulatory network is shown in red. Toxin-antitoxin (TA) systems stabilize cassette arrays in SCIs and supercoiling, replication (lagging strand template), and conjugation favor *attC* site folding and cassette dynamics. Representation of the connections between integrons and bacterial physiology is shown in green. Conjugation, competence, and antibiotics induce the SOS response and integrase expression, and carbon sources initiate catabolite repression and integrase expression.

Taken together, these results extend the list of processes intimately connecting the integron system with its host cell physiology (Fig. 6). This complex and extensive network of regulation processes constitutes a powerful and daunting system, making it increasingly difficult to limit the spread of multidrug resistance among bacteria.

## MATERIALS AND METHODS

### *In silico* analysis. (i) Data.

The sequences and annotations of complete genomes were downloaded from NCBI RefSeq (last accessed in November 2013, http://ftp.ncbi.nih.gov/genomes/refseq/bacteria/). Using the IntegronFinder program (https://github.com/gem-pasteur/Integron_Finder), we analyzed 2,484 bacterial genomes, including 2,626 replicons labeled as chromosomes and 2,006 replicons labeled as plasmids. IntegronFinder ensures an automatic and accurate identification of integrons, cassette arrays, and *attC* sites.

### (ii) SCI and MI classification.

Several criteria were used to determine the integron classification (10). Briefly, integrons were considered SCIs when they were present in the genomes of all the available sequenced strains of the species or when they contained more than 19 *attC* sites. They were considered MIs when they were absent in more than 40% of the sequenced genomes of the species, when they were present on a plasmid, or when the integrase was from one of the five classes of MIs.

### (iii) CDS analysis in sequenced strains containing integrons.

The set of MIs had 851 CDSs, whereas the set of SCIs had 2,856 CDSs, together belonging to 393 integrons. The replicons containing integrons had 472,225 CDSs. Leading and lagging strands were determined using the information from the OriC prediction database (48). The leading strand was defined as the strand with an increasing gradient of GC disparity (the GC disparity is a measure of the Z-curve representing the excess of G over C, a similar measure to the GC skew). The complementary strand was defined as the lagging strand.

### (iv) CDS analysis in *Vibrio* strains.

Annotated genomes of the indicated *Vibrio* strains were downloaded from NCBI RefSeq. CDS lengths were those from the RefSeq annotations. NCBI reference sequences of the genomes and position and length of integrases are presented in Table S1A in the supplemental material.

10.1128/mBio.02296-16.9 TABLE S1 (A) Accession numbers of the genomes and positions and lengths of integrases. (B) Bacterial strains. (C) Plasmids. (D) Primers and synthetic fragments (sequences are given in the 5′ → 3′ direction). Download TABLE S1, PDF file, 0.16 MB. Copyright © 2017 Loot et al. 2017 Loot et al. This content is distributed under the terms of the Creative Commons Attribution 4.0 International license.

### (v) *attC* sites used for the analysis.

For MI and SCI *attC* site comparison, we used the *attC* sites published and classified in reference 10 (185 different MI *attC* sites and 1,744 different sedentary CI *attC* sites). We reran IntegronFinder with the clustering parameters of 15 kb (-dt 15000 instead of 4 kb by default) for six known SCIs that IntegronFinder could not properly aggregate because of this threshold. The sequences of *attC* sites included the full 7-bp-long R box (the variable 4 bp of the R’ sequence not necessarily being complementary to their counterparts in the R” sequence, as is the case for *attC* sites in the integron array).

### (vi) *attC* site folding, Δ*G* and pfold predictions, and skews.

All folding predictions were obtained by RNAfold program from the ViennaRNA 2 package (49) with the set of DNA folding parameters derived from reference 50. We used the -p option to compute the partition functions and the -c option to add constraints required for a recombinogenic structure: pairing of the L’ and L’’ sequences and pairing of the 4 bases 5′-YAAC-3′ in the R’ sequence with the 4 bases 5′-GTTR-3′ in the R’’ sequence. We report the structures and Δ*G* values of the minimal free energy (MFE) structure. In order to obtain the pfold (probability of folding a recombinogenic structure) values, folding predictions were performed with and without constraints. pfold was calculated as the Boltzmann probability of the constrained (recombinogenic) structure in the ensemble: $\text{Pfold}\;=\;e^{\frac{E_{u}\;-\;E_{c}}{RT}}$, where *E*_*u*_ is the Gibbs free energy of the unconstrained (total) ensemble, *E*_*c*_ is the Gibbs free energy of the constrained (recombinogenic) ensemble, *R* is the gas constant, and *T* is the absolute temperature (51). GC skew measures the abundance of guanines (G) compared to cytosines (C) on the top strand: (G − C)/(G + C); AT skew measures the abundance of adenines (A) compared to thymines (T) on the top strand: (A − T)/(A + T).

### *In vivo* studies. (i) Bacterial strains and media.

Bacterial strains used in this study are described in Table S1B.

### (ii) Plasmids and primers.

Plasmids, primers, and synthetic fragments used in this study are described in Tables S1C and S1D.

### (iii) Integron cassette excision assay.

The pBAD::*intI1* plasmid (p3938) was introduced by transformation into the MG1655*ΔdapA* derivative strains containing insertions (in the *attB* lambda site) of plasmids carrying a *dapA* gene interrupted by the synthetic cassettes (Tables S1C and S1D). These strains are unable to synthesize 2,6-diaminopimelic acid (DAP), and as a result, they are not viable without DAP supplement in the medium. Recombination between *attC* sites causes excision of the synthetic cassette, restoring a functional *dapA* gene and allowing the strain to grow on DAP-free medium. After overnight growth in the presence of appropriate antibiotics (spectinomycin [Sp], carbenicillin [Carb]), DAP, and glucose, strains were cultivated for 6 h in the presence of the appropriate antibiotic (Carb), DAP, l-arabinose (Ara), and IPTG to allow *intI1* expression (Pbad) and *dapA* expression (P_*lac*_ promoter), respectively. Then, cultures were plated on agar containing either LB with IPTG and Sp or LB with DAP, IPTG, and Sp. Recombination activity was calculated as the ratio of the number of cells growing in the absence of DAP over the total number of cells. For each reaction, we confirmed cassette excision by performing PCRs with SW23begin/DapA-R primers generating products of 715, 763, 784, and 832 bp for *attC*_*aadA7*_-*attC*_*ereA2*_, *attC*_*aadA7*_-VCR_126_, VCR_126*_-VCR_126**_, and VCR_16*_-VCR_64_ cassettes, respectively.

## References

[1] Stokes HW, Hall RM 1989 A novel family of potentially mobile DNA elements encoding site-specific gene-integration functions: integrons. Mol Microbiol 3:1669–1683. doi:10.1111/j.1365-2958.1989.tb00153.x. 2560119

[2] Fluit AC, Schmitz FJ 2004 Resistance integrons and super-integrons. Clin Microbiol Infect 10:272–288. doi:10.1111/j.1198-743X.2004.00858.x. 15059115

[3] Mazel D 2006 Integrons: agents of bacterial evolution. Nat Rev Microbiol 4:608–620. doi:10.1038/nrmicro1462. 16845431

[4] Boucher Y, Cordero OX, Takemura A, Hunt DE, Schliep K, Bapteste E, Lopez P, Tarr CL, Polz MF 2011 Local mobile gene pools rapidly cross species boundaries to create endemicity within global Vibrio cholerae populations. mBio 2:e00335-10. doi:10.1128/mBio.00335-10. 21486909 PMC3073641

[5] Cambray G, Guerout AM, Mazel D 2010 Integrons. Annu Rev Genet 44:141–166. doi:10.1146/annurev-genet-102209-163504. 20707672

[6] Rapa RA, Labbate M 2013 The function of integron-associated gene cassettes in Vibrio species: the tip of the iceberg. Front Microbiol 4:385. doi:10.3389/fmicb.2013.00385. 24367362 PMC3856429

[7] Escudero JA, Loot C, Nivina A, Mazel D 2015 The integron: adaptation on demand. Microbiol Spectr 3:MDNA3-0019-2014. doi:10.1128/microbiolspec.MDNA3-0019-2014. 26104695

[8] Partridge SR, Tsafnat G, Coiera E, Iredell JR 2009 Gene cassettes and cassette arrays in mobile resistance integrons. FEMS Microbiol Rev 33:757–784. doi:10.1111/j.1574-6976.2009.00175.x. 19416365

[9] Naas T, Mikami Y, Imai T, Poirel L, Nordmann P 2001 Characterization of In53, a class 1 plasmid- and composite transposon-located integron of Escherichia coli which carries an unusual array of gene cassettes. J Bacteriol 183:235–249. doi:10.1128/JB.183.1.235-249.2001. 11114922 PMC94871

[10] Cury J, Jové T, Touchon M, Néron B, Rocha EP 2016 Identification and analysis of integrons and cassette arrays in bacterial genomes. Nucleic Acids Res 44:4539–4550. doi:10.1093/nar/gkw319. 27130947 PMC4889954

[11] Recchia GD, Hall RM 1995 Gene cassettes: a new class of mobile element. Microbiology 141:3015–3027. doi:10.1099/13500872-141-12-3015. 8574395

[12] Francia MV, Zabala JC, de la Cruz F, Garcia-Lobo JM 1999 The IntI1 integron integrase preferentially binds single-stranded DNA of the *attC* site. J Bacteriol 181:6844–6849. 10542191 10.1128/jb.181.21.6844-6849.1999 PMC94154

[13] Johansson C, Kamali-Moghaddam M, Sundström L 2004 Integron integrase binds to bulged hairpin DNA. Nucleic Acids Res 32:4033–4043. doi:10.1093/nar/gkh730. 15289577 PMC506814

[14] Bouvier M, Demarre G, Mazel D 2005 Integron cassette insertion: a recombination process involving a folded single strand substrate. EMBO J 24:4356–4367. doi:10.1038/sj.emboj.7600898. 16341091 PMC1356339

[15] Escudero JA, Loot C, Parissi V, Nivina A, Bouchier C, Mazel D 2016 Unmasking the ancestral activity of integron integrases reveals a smooth evolutionary transition during functional innovation. Nat Commun 7:10937. doi:10.1038/ncomms10937. 26961432 PMC4792948

[16] Bouvier M, Ducos-Galand M, Loot C, Bikard D, Mazel D 2009 Structural features of single-stranded integron cassette attC sites and their role in strand selection. PLoS Genet 5:e1000632. doi:10.1371/journal.pgen.1000632. 19730680 PMC2727003

[17] Nivina A, Escudero JA, Vit C, Mazel D, Loot C 2016 Efficiency of integron cassette insertion in correct orientation is ensured by the interplay of the three unpaired features of attC recombination sites. Nucleic Acids Res 44:7792–7803. doi:10.1093/nar/gkw646. 27496283 PMC5027507

[18] Frumerie C, Ducos-Galand M, Gopaul DN, Mazel D 2010 The relaxed requirements of the integron cleavage site allow predictable changes in integron target specificity. Nucleic Acids Res 38:559–569. doi:10.1093/nar/gkp990. 19914932 PMC2811028

[19] Stokes HW, O’Gorman DB, Recchia GD, Parsekhian M, Hall RM 1997 Structure and function of 59-base element recombination sites associated with mobile gene cassettes. Mol Microbiol 26:731–745. doi:10.1046/j.1365-2958.1997.6091980.x. 9427403

[20] Mazel D, Dychinco B, Webb VA, Davies J 1998 A distinctive class of integron in the *Vibrio cholerae* genome. Science 280:605–608. doi:10.1126/science.280.5363.605. 9554855

[21] Loot C, Ducos-Galand M, Escudero JA, Bouvier M, Mazel D 2012 Replicative resolution of integron cassette insertion. Nucleic Acids Res 40:8361–8370. doi:10.1093/nar/gks620. 22740653 PMC3458562

[22] Collis CM, Hall RM 1995 Expression of antibiotic resistance genes in the integrated cassettes of integrons. Antimicrob Agents Chemother 39:155–162. doi:10.1128/AAC.39.1.155. 7695299 PMC162502

[23] Baharoglu Z, Bikard D, Mazel D 2010 Conjugative DNA transfer induces the bacterial SOS response and promotes antibiotic resistance development through integron activation. PLoS Genet 6:e1001165. doi:10.1371/journal.pgen.1001165. 20975940 PMC2958807

[24] Hocquet D, Llanes C, Thouverez M, Kulasekara HD, Bertrand X, Plésiat P, Mazel D, Miller SI 2012 Evidence for induction of integron-based antibiotic resistance by the SOS response in a clinical setting. PLoS Pathog 8:e1002778. doi:10.1371/journal.ppat.1002778. 22719259 PMC3375312

[25] Barraud O, Ploy MC 2015 Diversity of class 1 integron gene cassette rearrangements selected under antibiotic pressure. J Bacteriol 197:2171–2178. doi:10.1128/JB.02455-14. 25897031 PMC4455274

[26] Guerin E, Cambray G, Sanchez-Alberola N, Campoy S, Erill I, Da Re S, Gonzalez-Zorn B, Barbé J, Ploy MC, Mazel D 2009 The SOS response controls integron recombination. Science 324:1034. doi:10.1126/science.1172914. 19460999

[27] Cambray G, Sanchez-Alberola N, Campoy S, Guerin E, Da Re S, González-Zorn B, Ploy MC, Barbé J, Mazel D, Erill I 2011 Prevalence of SOS-mediated control of integron integrase expression as an adaptive trait of chromosomal and mobile integrons. Mob DNA 2:6. doi:10.1186/1759-8753-2-6. 21529368 PMC3108266

[28] Baharoglu Z, Krin E, Mazel D 2012 Connecting environment and genome plasticity in the characterization of transformation-induced SOS regulation and carbon catabolite control of the Vibrio cholerae integron integrase. J Bacteriol 194:1659–1667. doi:10.1128/JB.05982-11. 22287520 PMC3302476

[29] Bikard D, Loot C, Baharoglu Z, Mazel D 2010 Folded DNA in action: hairpin formation and biological functions in prokaryotes. Microbiol Mol Biol Rev 74:570–588. doi:10.1128/MMBR.00026-10. 21119018 PMC3008174

[30] Loot C, Parissi V, Escudero JA, Amarir-Bouhram J, Bikard D, Mazel D 2014 The integron integrase efficiently prevents the melting effect of Escherichia coli single-stranded DNA-binding protein on folded attC sites. J Bacteriol 196:762–771. doi:10.1128/JB.01109-13. 24296671 PMC3911172

[31] Loot C, Bikard D, Rachlin A, Mazel D 2010 Cellular pathways controlling integron cassette site folding. EMBO J 29:2623–2634. doi:10.1038/emboj.2010.151. 20628355 PMC2928681

[32] Wolfson J, Dressler D 1972 Regions of single-stranded DNA in the growing points of replicating bacteriophage T7 chromosomes. Proc Natl Acad Sci U S A 69:2682–2686. doi:10.1073/pnas.69.9.2682. 4560695 PMC427016

[33] Moura A, Soares M, Pereira C, Leitão N, Henriques I, Correia A 2009 INTEGRALL: a database and search engine for integrons, integrases and gene cassettes. Bioinformatics 25:1096–1098. doi:10.1093/bioinformatics/btp105. 19228805

[34] Sigel A, Operschall BP, Sigel H 2014 Comparison of the pi-stacking properties of purine versus pyrimidine residues. Some generalizations regarding selectivity. J Biol Inorg Chem 19:691–703. doi:10.1007/s00775-013-1082-5. 24464134

[35] Rowe-Magnus DA, Guerout AM, Ploncard P, Dychinco B, Davies J, Mazel D 2001 The evolutionary history of chromosomal super-integrons provides an ancestry for multiresistant integrons. Proc Natl Acad Sci U S A 98:652–657. doi:10.1073/pnas.98.2.652. 11209061 PMC14643

[36] Gillings MR, Holley MP, Stokes HW, Holmes AJ 2005 Integrons in Xanthomonas: a source of species genome diversity. Proc Natl Acad Sci U S A 102:4419–4424. doi:10.1073/pnas.0406620102. 15755815 PMC555480

[37] Poirel L, Naas T, Nordmann P 2010 Diversity, epidemiology, and genetics of class D beta-lactamases. Antimicrob Agents Chemother 54:24–38. doi:10.1128/AAC.01512-08. 19721065 PMC2798486

[38] Postow L, Hardy CD, Arsuaga J, Cozzarelli NR 2004 Topological domain structure of the Escherichia coli chromosome. Genes Dev 18:1766–1779. doi:10.1101/gad.1207504. 15256503 PMC478196

[39] Shlyakhtenko LS, Hsieh P, Grigoriev M, Potaman VN, Sinden RR, Lyubchenko YL 2000 A cruciform structural transition provides a molecular switch for chromosome structure and dynamics. J Mol Biol 296:1169–1173. doi:10.1006/jmbi.2000.3542. 10698623

[40] Johnson A, O’Donnell M 2005 Cellular DNA replicases: components and dynamics at the replication fork. Annu Rev Biochem 74:283–315. doi:10.1146/annurev.biochem.73.011303.073859. 15952889

[41] Ton-Hoang B, Pasternak C, Siguier P, Guynet C, Hickman AB, Dyda F, Sommer S, Chandler M 2010 Single-stranded DNA transposition is coupled to host replication. Cell 142:398–408. doi:10.1016/j.cell.2010.06.034. 20691900 PMC2919506

[42] Soler-Bistué A, Mondotte JA, Bland MJ, Val ME, Saleh MC, Mazel D 2015 Genomic location of the major ribosomal protein gene locus determines Vibrio cholerae global growth and infectivity. PLoS Genet 11:e1005156. doi:10.1371/journal.pgen.1005156. 25875621 PMC4395360

[43] Val ME, Marbouty M, de Lemos Martins F, Kennedy SP, Kemble H, Bland MJ, Possoz C, Koszul R, Skovgaard O, Mazel D 2016 A checkpoint control orchestrates the replication of the two chromosomes of Vibrio cholerae. Sci Adv 2:e1501914. doi:10.1126/sciadv.1501914. 27152358 PMC4846446

[44] Feng L, Reeves PR, Lan R, Ren Y, Gao C, Zhou Z, Ren Y, Cheng J, Wang W, Wang J, Qian W, Li D, Wang L 2008 A recalibrated molecular clock and independent origins for the cholera pandemic clones. PLoS One 3:e4053. doi:10.1371/journal.pone.0004053. 19115014 PMC2605724

[45] Chun J, Grim CJ, Hasan NA, Lee JH, Choi SY, Haley BJ, Taviani E, Jeon YS, Kim DW, Lee JH, Brettin TS, Bruce DC, Challacombe JF, Detter JC, Han CS, Munk AC, Chertkov O, Meincke L, Saunders E, Walters RA, Huq A, Nair GB, Colwell RR 2009 Comparative genomics reveals mechanism for short-term and long-term clonal transitions in pandemic Vibrio cholerae. Proc Natl Acad Sci U S A 106:15442–15447. doi:10.1073/pnas.0907787106. 19720995 PMC2741270

[46] Rowe-Magnus DA, Guerout AM, Mazel D 2002 Bacterial resistance evolution by recruitment of super-integron gene cassettes. Mol Microbiol 43:1657–1669. doi:10.1046/j.1365-2958.2002.02861.x. 11952913

[47] Engelstädter J, Harms K, Johnsen PJ 2016 The evolutionary dynamics of integrons in changing environments. ISME J 10:1296–1307. doi:10.1038/ismej.2015.222. 26849314 PMC5029196

[48] Gao F, Luo H, Zhang CT 2013 DoriC 5.0: an updated database of oriC regions in both bacterial and archaeal genomes. Nucleic Acids Res 41:D90–D93. doi:10.1093/nar/gks990. 23093601 PMC3531139

[49] Lorenz R, Bernhart SH, Höner Zu Siederdissen C, Tafer H, Flamm C, Stadler PF, Hofacker IL 2011 ViennaRNA package 2.0. Algorithms Mol Biol 6:26. doi:10.1186/1748-7188-6-26. 22115189 PMC3319429

[50] Mathews DH, Disney MD, Childs JL, Schroeder SJ, Zuker M, Turner DH 2004 Incorporating chemical modification constraints into a dynamic programming algorithm for prediction of RNA secondary structure. Proc Natl Acad Sci U S A 101:7287–7292. doi:10.1073/pnas.0401799101. 15123812 PMC409911

[51] Hofacker IL, Lorenz R 2013 Predicting RNA structure: advances and limitations. Methods Mol Biol 1086:1–19. doi:10.1007/978-1-62703-667-2_1. 24136595

