# Supplementary material for: Differences in Integron Cassette Excision Dynamics Shape a Trade-Off between Evolvability and Genetic Capacitance
Source: mBio. 2017 Mar 28;8(2):e02296-16. doi: 10.1128/mBio.02296-16 (PMC5371416; doi:10.1128/mBio.02296-16)
Supplement: FIG S3 [file mbo002173252sf3.pdf]

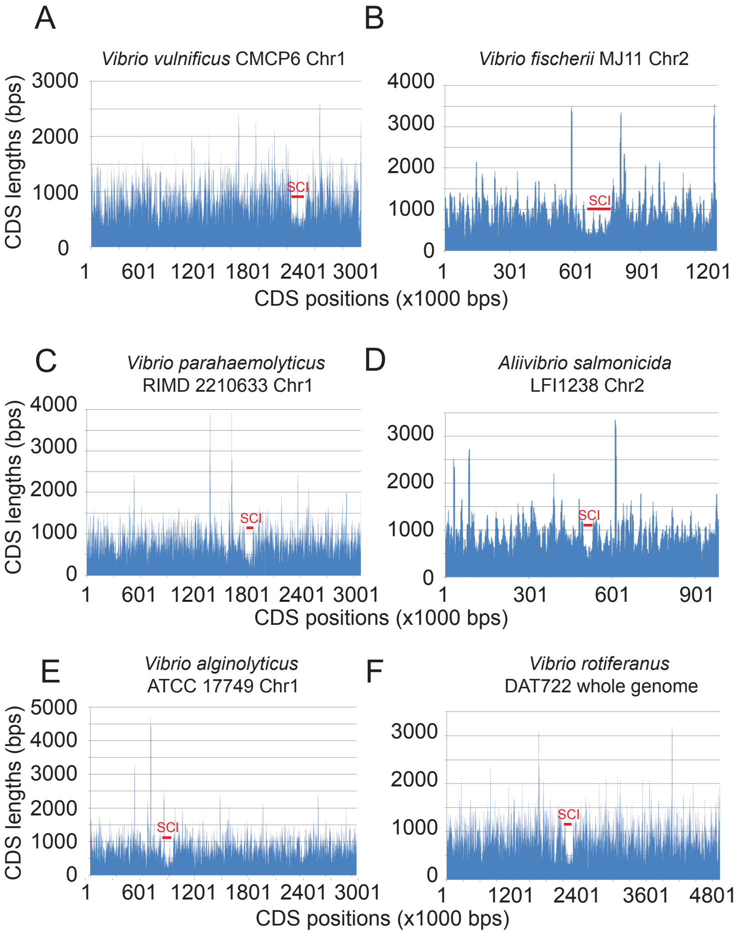
A
Vibrio vulnificus CMCP6 Chr1
B
Vibrio fischerii MJ11 Chr2
C
Vibrio parahaemolyticus RIMD 2210633 Chr1
D
Aliivibrio salmonicida LFI1238 Chr2
E
Vibrio alginolyticus ATCC 17749 Chr1
F
Vibrio rotiferanus DAT722 whole genome
SCI
CDS lengths (bps)
CDS positions (x1000 bps)
