# Supplementary figures and images for: Differences in Integron Cassette Excision Dynamics Shape a Trade-Off between Evolvability and Genetic Capacitance
Source: mBio. 2017 Mar 28;8(2):e02296-16. doi: 10.1128/mBio.02296-16 (PMC5371416; doi:10.1128/mBio.02296-16)

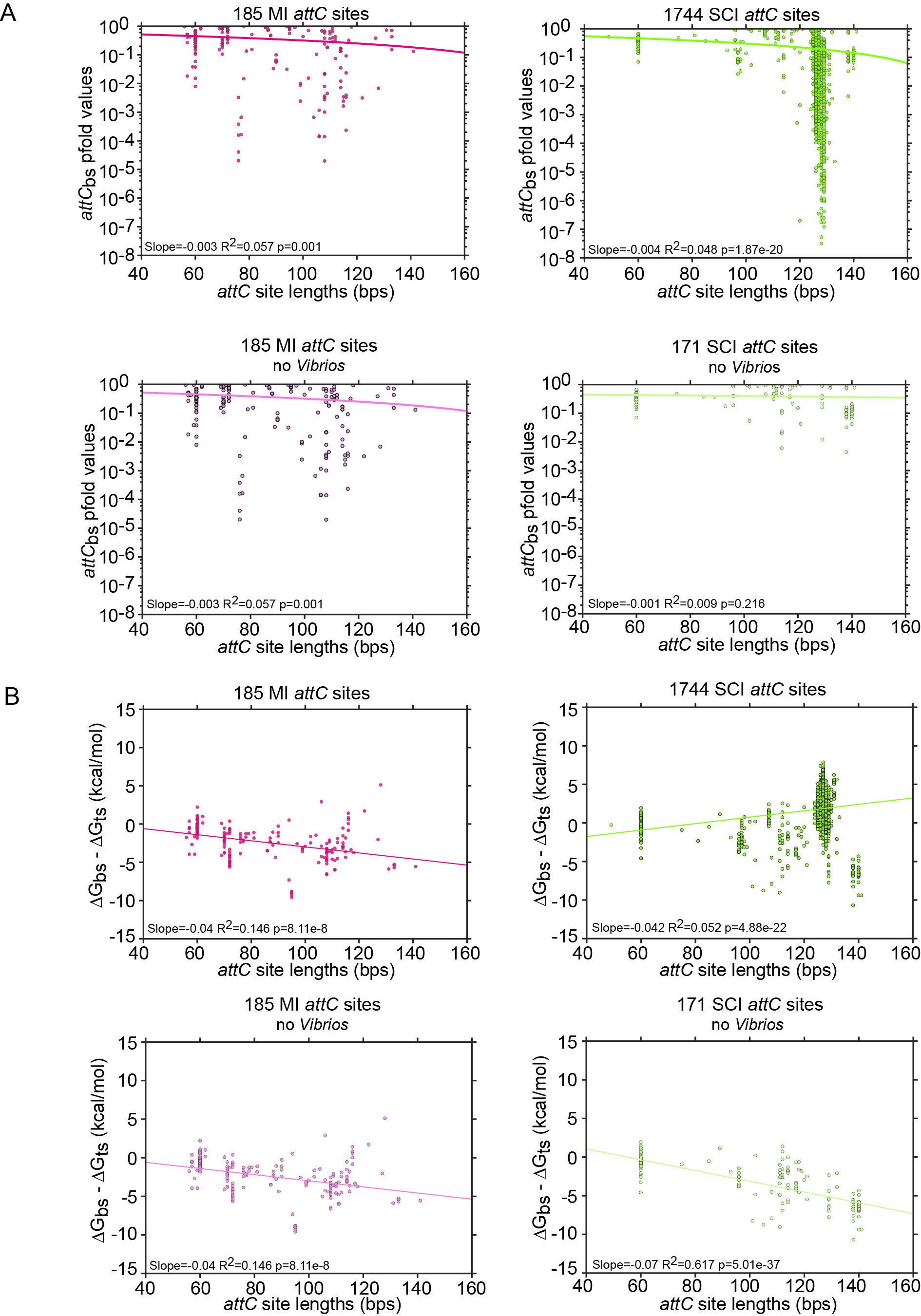

Supplement: FIG S5 [file mbo002173252sf5.jpg]
