# Supplementary material for: Differences in Integron Cassette Excision Dynamics Shape a Trade-Off between Evolvability and Genetic Capacitance
Source: mBio. 2017 Mar 28;8(2):e02296-16. doi: 10.1128/mBio.02296-16 (PMC5371416; doi:10.1128/mBio.02296-16)
Supplement: FIG S6 [file mbo002173252sf6.pdf]

***attC*$_{aadA7}$ - *attC*$_{ereA2}$ synthetic cassettes**

***attC*$_{aadA7}$**

top strand (ts) sequence:

5’-TTATAACAATTCATTCAAGCCGACGCCGCTTCGCGGCGCGGCTTAATTCAAGCGTTAGAT

ΔG_{bs} value of the most stable structure without constraint= -15.5 kcal/mol

ΔG_{bs} value of the most stable structure with constraints (recombinogenic)= -15.5 kcal/mol

attC$_{bs}$ pfold value calculated with constrained vs. unconstrained folding= 0.30

The most stable structure is the recombinogenic one:

attC$_{aadA7}$bs

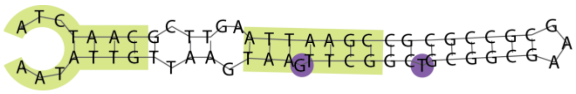

***attC*$_{ereA2}$**

ts sequence: 5’-ATCTAACCTGCCAATCCACCGGACGGTTTTCAACCGCCGGTGATCAGCGCGTTATAC

ΔG_{bs} value of the most stable structure without constraints= -14.0 kcal/mol

ΔG_{bs} value of the most stable structure with constraints (recombinogenic)= -14.0 kcal/mol

attC$_{bs}$ pfold value calculated with constrained vs. unconstrained folding= 0.44

The most stable structure is the recombinogenic one:

attC$_{ereA2}$bs

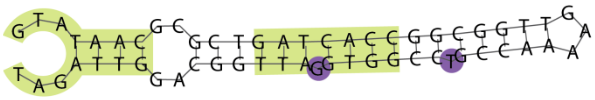

***attC*$_{aadA7}$ - *VCR*$_{126}$ synthetic cassettes**

***attC*$_{aadA7}$**

ts sequence: 5’-TTATAACAATTCATTCAAGCCGACGCCGCTTCGCGGCGCGGCTTAATTCAAGCGTTAGAT

ΔG_{bs} value of the most stable structure without constraints= -15.5 kcal/mol

ΔG_{bs} value of the most stable structure with constraints (recombinogenic)= -15.5 kcal/mol

attC$_{bs}$ pfold value calculated with constrained vs. unconstrained folding= 0.30

The most stable structure is the recombinogenic one:

attC$_{aadA7}$bs

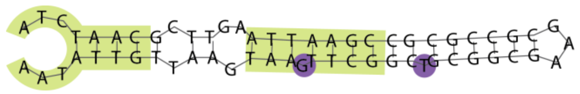

VCR$_{126}$

ts sequence: 5'- ATCTAACAAACGCCTCAAGAGGGACTGTCAACGCGTGGCGTTTCCAGTCCCATTGAGCCG CGGTGGTTGCTGTTGTTGTGTTTGAGTTTAGTGGTAGTGCGTTGTCAGCCCCTTAGGCGGGCGTTATAC

ΔG_{bs} value of the most stable structure without constraints= -24.1 kcal/mol

ΔG_{bs} value of the most stable structure with constraints (recombinogenic)= -16.7 kcal/mol

attC$_{bs}$ pfold value calculated with constrained vs. unconstrained folding= 1.04×10^{-5}

The most stable structure is a non-recombinogenic one (improperly folded).

The recombinogenic structure is:

VCR$_{126}$bs

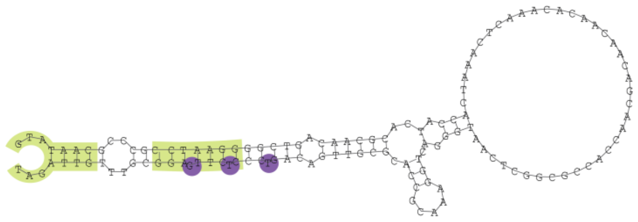

***VCR$_{126*}$* - *VCR$_{126**}$* synthetic cassettes**

VCR$_{126*}$

ts sequence: 5'- TTATAACAAACGCCTCAAGAGGGACTGTCAACGCGTGGCGTTTCCAGTCCCATTGGCCGC

GGTGGTTGCTGTTGTTGTGTTTGAGTTTAGTGGTAGTGCGTTGTCAGCCCCTTAGGCGGGCGTTAGAT

ΔG_{bs} value of the most stable structure without constraints= -20.4 kcal/mol

ΔG_{bs} value of the most stable structure with constraints (recombinogenic)= -16.7 kcal/mol

attC$_{bs}$ pfold value calculated with constrained vs. unconstrained folding= 3.09×10^{-3}

The most stable structure is a non-recombinogenic one (improperly folded).

The recombinogenic structure is:

VCR$_{126*}$bs

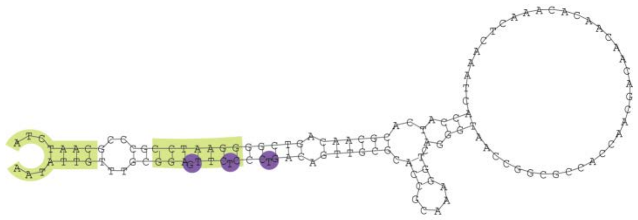

VCR$_{126**}$

ts sequence: 5'- ATCTAACAAACGCCTCAAGAGGGACTGTCAACGCGTGGCGTTTCCAGTCCCATTGAGCCG CGGTGGTTGCTGTTGTTGTGTTTGAGTTTAGTGGTAGTGCGTTGTCAGCCCCTTAGGCGGGCGTTATTA

ΔG_{bs} value of the most stable structure without constraints= -24.1 kcal/mol

ΔG_{bs} value of the most stable structure with constraints (recombinogenic)= -16.7 kcal/mol

attC$_{bs}$ pfold value calculated with constrained vs. unconstrained folding= 7.86×10^{-6}

The most stable structure is a non-recombinogenic one (improperly folded).

The recombinogenic structure is:

VCR$_{126**}$bs

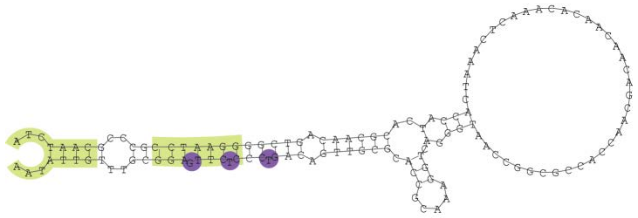

***VCR$_{16*}$* - *VCR$_{64}$* synthetic cassettes**

VCR_{16*}

ts sequence: 5’- TTATAACAAACGGCTCAAGAGGGACTGTCAACGCGTGGCGTTTCCAGTCCCAAGAACTGC GGCGTTTACGGTTGCTGTGTTTGAGTTTAGTGTTAATGCGTTGCCAGCCCCTTAGCCGGGCGTTAGAT

ΔG_{bs} value of the most stable structure without constraints= -19.6 kcal/mol

ΔG_{bs} value of the most stable structure with constraints (recombinogenic)= -19.6 kcal/mol

attC$_{bs}$ pfold value calculated with constrained vs. unconstrained folding= 0.35

The most stable structure is the recombinogenic one:

$VCR_{16*}bs$

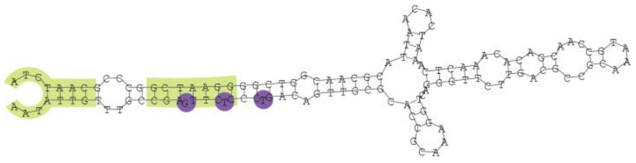

VCR_{64}

ts sequence: 5’- ATCTAACAAACGCCTTAAGAGGGACTGCCAACGCGTGGCATTTCCAGTCCCAATGAGCCG TGGTGGTTACGGTTGTTGTGTTTGAGTTTCGTTGTTATGCGTTGTCAGCCCCTTAGGCGGGCGTTATTA

ΔG_{bs} value of the most stable structure without constraints= -19.8 kcal/mol

ΔG_{bs} value of the most stable structure with constraints (recombinogenic)= -19.8 kcal/mol

attC$_{bs}$ pfold value calculated with constrained vs. unconstrained folding= 0.24

The most stable structure is the recombinogenic one:

$VCR_{64}bs$

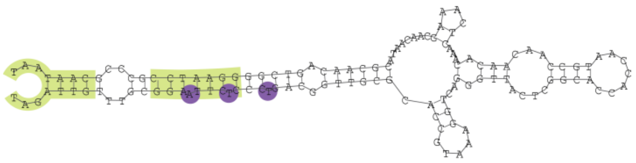

Structures were determined by the RNAfold program from the ViennaRNA 2 package (Methods).
