# Supplementary material for: Differences in Integron Cassette Excision Dynamics Shape a Trade-Off between Evolvability and Genetic Capacitance
Source: mBio. 2017 Mar 28;8(2):e02296-16. doi: 10.1128/mBio.02296-16 (PMC5371416; doi:10.1128/mBio.02296-16)
Supplement: TABLE S1 [file mbo002173252st1.pdf]

Table S1A

Vibrio spp. strains	NCBI reference sequence	Integrase gene position	Integrase gene length (bps)
Aliivibrio salmonicida LFI1238	NC_011312<br>NC_011313	593499-594482	984_reverse
Vibrio alginolitycus ATCC 17749	NC_022349<br>NC_022359	1009839-1010801	963_forward
Vibrio cholerae El Tor strain N16961	NC_002505<br>NC_002506	309750-310712	963_reverse
Vibrio fischerii MJ11	NC_011184<br>NC_011186	817245-818228	984_forward
Vibrio parahaemolyticus RIMD 2210633	NC_004603<br>NC_004605	1948281-1949243	963_forward
Vibrio rotiferianus DAT722	NZ_AFAJ00000000<br>NZ_AJZL00000000	2363443-2364405	936_reverse
Vibrio vulnificus CMCP6	NC_004459<br>NC_004460	2438314-2439276	963_reverse

Table S1B

**Basic strains***		
**Strain number**	**Relevant *Escherichia coli* genotypes and description**	**References**
ω9420	**Π1** DH5α Δ*thyA*::*erm-pir116*) [Erm^R]	*Demarre et al, 2005*
ω8486	**MG1655**Δ*dapA recA269::tn10* [Tc^R]	*Loot et al, 2014*
ω8488	ω8486 transformed with p3153 [Tc^R, Carb^R]	*Loot et al, 2014*

Strain number	Relevant *Vibrio cholerae* genotype and description	References
ω8637	**N16961** [Str^R]	Heidelberg *et al*, 2000

**Derivative** MG1655Δ*dapA recA269::tn10* **strains*,****			
**Cassette description**	**Plasmid number**	**Strain number (inserted plasmid)**	**Strain number (inserted plasmid+ pBAD*intI1*)**
$attC_{aadA7}$-60bp-$attC_{ereA2}$ (attP+)	pB322	C188	C448
$attC_{aadA7}$-60+**400bp*lacZ***-$attC_{ereA2}$ (attP+)	pB429	C189	C316
$attC_{aadA7}$-60+**750bp*lacZ***-$attC_{ereA2}$ (attP+)	pB254	D004	D066
$attC_{aadA7}$-60+**1500bp*lacZ***-$attC_{ereA2}$ (attP+)	pB255	B552	C480
$attC_{aadA7}$-60+**3000bp*lacZ***-$attC_{ereA2}$ (attP+)	pB321	E109	E156
$attC_{aadA7}$-60bp-$attC_{ereA2}$ (attP-)	pB256	C186	C318
$attC_{aadA7}$-60+**400bp*lacZ***-$attC_{ereA2}$ (attP-)	pB428	B548	C319
$attC_{aadA7}$-60+**750bp*lacZ***-$attC_{ereA2}$ (attP-)	pB257	D005	D067
$attC_{aadA7}$-60+**1500bp*lacZ***-$attC_{ereA2}$ (attP-)	pB253	E108	E154
$attC_{aadA7}$-60+**3000bp*lacZ***-$attC_{ereA2}$ (attP-)	pB252	B550	C437
$attC_{aadA7}$-60+**750bp*hubP***-$attC_{ereA2}$ (attP+)	pE511	E906	F299
$attC_{aadA7}$-60+**1500bp*hubP***-$attC_{ereA2}$ (attP+)	pE512	F052	F300
$attC_{aadA7}$-60+**3000bp*hubP***-$attC_{ereA2}$ (attP+)	pE513	E908	F301
$attC_{aadA7}$-60bp-VCR_{126} (attP+)	pA621	D098	D115

$attC_{aadA7}$-60+**400bp*lacZ***-VCR$_{126}$ (attP+)	pB332	E288	E297
$attC_{aadA7}$-60+**750bp*lacZ***-VCR$_{126}$ (attP+)	pA696	E291	E300
$attC_{aadA7}$-60+**1500bp*lacZ***-VCR$_{126}$ (attP+)	pA703	B547	C449
$attC_{aadA7}$-60+**3000bp*lacZ***-VCR$_{126}$ (attP+)	pA705	C185	C310
$attC_{aadA7}$-60bp-VCR$_{126}$ (attP-)	pA672	E189	E310
$attC_{aadA7}$-60+**400bp*lacZ***-VCR$_{126}$ (attP-)	pB331	B541	C312
$attC_{aadA7}$-60+**750bp*lacZ***-VCR$_{126}$ (attP-)	pA669	E195	E316
$attC_{aadA7}$-60+**1500bp*lacZ***-VCR$_{126}$ (attP-)	pA675	E187	E308
$attC_{aadA7}$-60+**3000bp*lacZ***-VCR$_{126}$ (attP-)	pA670	B544	C315
$attC_{aadA7}$-60+**750bp*hubP***-VCR$_{126}$ (attP+)	pE507	E902	F302
$attC_{aadA7}$-60+**1500bp*hubP***-VCR$_{126}$ (attP+)	pE508	E903	F303
$attC_{aadA7}$-60+**3000bp*hubP***-VCR$_{126}$ (attP+)	pE509	E904	F304
$attC_{aadA7}$-60+**750bp*hubP***-VCR$_{126}$ (attP-)	pE503	E898	F305
$attC_{aadA7}$-60+**1500bp*hubP***-VCR$_{126}$ (attP-)	pE504	E899	F306
$attC_{aadA7}$-60+**3000bp*hubP***-VCR$_{126}$ (attP-)	pE505	F045	F307
VCR$_{16*}$-60bp-VCR$_{64}$ (attP+)	pA702	D087	D107
VCR$_{16*}$-60+**400bp*lacZ***-VCR$_{64}$ (attP+)	pB328	D090	D110
VCR$_{16*}$-60+**750bp*lacZ***-VCR$_{64}$ (attP+)	pA717	D088	D108
VCR$_{16*}$-60+**1500bp*lacZ***-VCR$_{64}$ (attP+)	pA701	D094	D114
VCR$_{16*}$-60+**3000bp*lacZ***-VCR$_{64}$ (attP+)	pA697	D093	D113
VCR$_{16*}$-60bp-VCR$_{64}$ (attP-)	pA671	D086	D106
VCR$_{16*}$-60+**400bp*lacZ***-VCR$_{64}$ (attP-)	pB327	D089	D109
VCR$_{16*}$-60+**750bp*lacZ***-VCR$_{64}$ (attP-)	pA667	D085	D105

VCR$_{16*}$-60+**1500bp*lacZ***-VCR$_{64}$ (attP-)	pA673	D092	D112
VCR$_{16*}$-60+**3000bp*lacZ***-VCR$_{64}$ (attP-)	pA663	D091	D111
VCR$_{126*}$-60bp-VCR$_{126**}$ (attP+)	pE894	F059	F294
VCR$_{126*}$-60+**400bp*lacZ***-VCR$_{126**}$ (attP+)	pE978	F060	F295
VCR$_{126*}$-60+**750bp*lacZ***-VCR$_{126**}$ (attP+)	pF308	F155	F296
VCR$_{126*}$-60+**1500bp*lacZ***-VCR$_{126**}$ (attP+)	pE980	F062	F297
VCR$_{126*}$-60+**3000bp*lacZ***-VCR$_{126**}$ (attP+)	pE922	F063	F298
VCR$_{126*}$-60bp-VCR$_{126**}$ (attP-)	pE893	F054	F289
VCR$_{126*}$-60+**400bp*lacZ***-VCR$_{126**}$ (attP-)	pE975	F055	F290
VCR$_{126*}$-60+**750bp*lacZ***-VCR$_{126**}$ (attP-)	pF309	F156	F291
VCR$_{126*}$-60+**1500bp*lacZ***-VCR$_{126**}$ (attP-)	pE977	F057	F292
VCR$_{126*}$-60+**3000bp*lacZ***-VCR$_{126**}$ (attP-)	pE921	F058	F293

*****_Escherichia coli_ and _Vibrio cholerae_ strains were grown in Luria Bertani (LB) broth at 37°C or 30°C (when specified). Antibiotics were used at the following concentrations: carbenicillin (Carb), 100 µg/ml, spectinomycin (Sp), 40 µg/ml, erythromycin (Erm), 200 µg/ml, tetracyclin (Tet), 15 µg/ml, streptomycin (Str), 200 µg/ml, kanamycin (Kan), 25 µg/ml, Thymidine (Thy) and diaminopimelic acid (DAP) were supplemented when necessary to a final concentration of 0.3mM. To induce the P_{bad} promoter, L-arabinose (Ara) was added to a final concentration of 2mg/ml; to repress it, glucose (Glc) was added to a final concentration of 10mg/ml. To induce the P_{lac} promoter, isopropyl-β-D-thiogalactopyranoside (IPTG) was added to a final concentration of 200µg/ml.

******All the constructed pSW derivative plasmids were inserted into the λ _attB_ site of the ω8488

chromosome through lambda recombination mediated by plasmid pTSA29-CXI (p3153, $Carb^R$, Valens & al., 2004). By inverting the *attP* site on the pSW plasmid, we inserted plasmids in both orientations: attP- corresponding to the bottom strand of *attC* sites carried by the lagging strand template, and attP+ to the bottom strand carried by the leading strand template. We confirmed plasmid insertions by performing PCR reactions with o1897/o1898 (presence of a monomeric inserted plasmid) and o361/o3725 (plasmid insertion in *attB* site) primers. We performed all the experiments in *recA* deleted strains to avoid homologous recombination between the *lacZ* fragment in the synthetic cassettes and the endogenous *lacZ* gene, as well as between *attC* sites having high degree of identity (i.e: VCR sites).

Table S1C

**Plasmids used in this study***		
**Plasmid name and description**	**Plasmid number**	**References**
pBADIntI1, $ori_{ColE1,}$ [$Carb^R$]	p3938	Demarre *et al*, 2007
pTSA29-CXI, $ori_{pSC101,}$ [$Carb^R$]	p3153	Valens *et al*, 2004
pSW23T, ori_{R6K}, [Sp^R]	p7988	Laboratory collection
pSU38Δ::*lacZ*::*attC*$_{aadA7}$-*catT4t*-VCR_2, *ori*$_{p15A,}$ [Kan]^R	p1372	Demarre *et al*, 2007
pRS551, ori_{pMB1}, [$Carb^R$, Kan^R]	p923	Simons *et al*, 1987
pSW*attI*-*attC*$_{aadA7}$-*dapA*, [Cm^R]	p7923	Baharoglu *et al*, 2010
pMA-VCR_{126*}-term, ori_{ColE1}, [$Carb^R$]	p8208	VCR_{126*}-term GeneArt fragment (see Table III) in pMA plasmid

pTOPO-VCR_{16*}-VCR_{64}, ori_{PUC}, $[Kan^R]$	pE884	**VCR_{16*}-VCR_{64} gBlocks® Gene Fragment from Integrated DNA Technologies (see Table III) in** pCR™-Blunt II-TOPO^R (Invitrogen)

**Derivative** pSW **plasmids constructed in this study**		
**Plasmid name and description**<br>pSW23T, ori_{R6K}, $[Sp^R]$	**Plasmid number**	**Relevant properties and construction**
pSW*attC*$_{aadA7}$-VCR_{126} (attP+)	p8146	**Plac-*attC*$_{aadA7}$** fragment (amplification of p1372 using o299 and o845 followed by EcoRI/BamHI digestion) and **VCR_{126}** fragment (annealing between o876, o877, o878 and o879) in EcoRI/XmaI digested p7988
pSW*attC*$_{aadA7}$-VCR_{126**} (attP+)	p8159	***attC*$_{aadA7}$** fragment (p8146 digested by EcoRI/BglI) and **VCR_{126**}** fragment (annealing between o885 and o886) in EcoRI/XmaI digested p7988
pSW*attC*$_{aadA7}$-60bp-VCR_{126} (attP+)	p8161	**Terminator** fragment (annealing between o883 and o884) in BamHI digested p8146
pSWVCR_{126*}-60bp-VCR_{126**} (attP+)	p8241	**VCR_{126*}-term** fragment (XbaI/BglII digested p8208) in XbaI/BamHI digested p8159
pSW*attC*$_{aadA7}$-60bp-VCR_{126} (attP-)	p8257	Inversion of the **attP** region of p8161 by SacI

		digestion
pSWVCR$_{126*}$-60bp-VCR$_{126**}$ (attP-)	p8256	Inversion of the **attP** region of p8241 by SacI digestion
pSW*attC$_{aadA7}$*-60+**3000bp*lacZ***-VCR$_{126}$ (attP+)	p8238	**3000bp*lacZ*** fragment (PCR fragment (o863 and o864) from p923)) in BamHI digested p8161
pSWVCR$_{126*}$-60+**3000bp*lacZ***-VCR$_{126**}$ (attP+)	p8265	**3000bp*lacZ*** fragment in BamHI digested p8241
pSW*attC$_{aadA7}$*-60+**3000bp*lacZ***-VCR$_{126}$ (attP-)	p8300	**3000bp*lacZ*** fragment in BamHI digested p8257
pSWVCR$_{126*}$-60+**3000bp*lacZ***-VCR$_{126**}$ (attP-)	p8298	**3000bp*lacZ*** fragment in BamHI digested p8256
pSW*attC$_{aadA7}$*-60+**750bp*lacZ***-VCR$_{126}$ (attP+)	p8255	**750bp*lacZ*** fragment (PCR fragment (o863 and o868) from p923)) in BamHI/NheI digested p8238
pSWVCR$_{126*}$-60+**750bp*lacZ***-VCR$_{126**}$ (attP+)	p8338	**750bp*lacZ*** fragment in BamHI/NheI digested p8265
pSW*attC$_{aadA7}$*-60+**750bp*lacZ***-VCR$_{126}$ (attP-)	p8341	**750bp*lacZ*** fragment in BamHI/NheI digested p8300
pSWVCR$_{126*}$-60+**750bp*lacZ***-VCR$_{126**}$ (attP-)	p8339	**750bp*lacZ*** fragment in BamHI/NheI digested p8298
pSW*attC$_{aadA7}$*-60bp-VCR$_{126}$ (attP+)	p9935	***dapA*** fragment (PCR fragment (o1069 and 1070) from p7923) in SmaI/PstI digested p8161
pSWVCR$_{126*}$-60bp-VCR$_{126**}$ (attP+)	p9936	***dapA*** fragment in SmaI/PstI digested p8241

pSW*attC*$_{aadA7}$-60bp-VCR$_{126}$ (attP-)	p9938	***dapA*** fragment in SmaI/PstI digested p8257
pSWVCR$_{126*}$-60bp-VCR$_{126**}$ (attP-)	p9937	***dapA*** fragment in SmaI/PstI digested p8256
pSW*attC*$_{aadA7}$-60+**3000bp*lacZ***-VCR$_{126}$ (attP+)	p8456	***dapA*** fragment in SmaI/PstI digested p8238
pSWVCR$_{126*}$-60+**3000bp*lacZ***-VCR$_{126**}$ (attP+)	p8459	***dapA*** fragment in SmaI/PstI digested p8265
pSW*attC*$_{aadA7}$-60+**3000bp*lacZ***-VCR$_{126}$ (attP-)	p8469	***dapA*** fragment in SmaI/PstI digested p8300
pSWVCR$_{126*}$-60+**3000bp*lacZ***-VCR$_{126**}$ (attP-)	p8467	***dapA*** fragment in SmaI/PstI digested p8298
pSW*attC*$_{aadA7}$-60+**750bp*lacZ***-VCR$_{126}$ (attP+)	p8458	***dapA*** fragment in SmaI/PstI digested p8255
pSWVCR$_{126*}$-60+**750bp*lacZ***-VCR$_{126**}$ (attP+)	p8460	***dapA*** fragment in SmaI/PstI digested p8338
pSW*attC*$_{aadA7}$-60+**750bp*lacZ***-VCR$_{126}$ (attP-)	p8463	***dapA*** fragment in SmaI/PstI digested p8341
pSWVCR$_{126*}$-60+**750bp*lacZ***-VCR$_{126**}$ (attP-)	p8461	***dapA*** fragment in SmaI/PstI digested p8339
pSW*attC*$_{aadA7}$-60+**1500bp*lacZ***-VCR$_{126}$ (attP+)	pA350	**1500bp*lacZ*** fragment (PCR fragment (o863 and o866) from p923)) in BamHI/NheI digested p8458
pSWVCR$_{126*}$-60+**1500bp*lacZ***-VCR$_{126**}$ (attP+)	pA351	**1500bp*lacZ*** fragment in BamHI/NheI digested p8460

pSW*attC*$_{aadA7}$-60+**1500bp*lacZ***-VCR$_{126}$ (attP-)	pA354	**1500bp*lacZ*** fragment in BamHI/NheI digested p8463
pSWVCR$_{126*}$-60+**1500bp*lacZ***-VCR$_{126**}$ (attP-)	pA352	**1500bp*lacZ*** fragment in BamHI/NheI digested p8461
pSW*attC*$_{aadA7}$-60bp-VCR$_{126}$ (attP+)	pA621	**FRT** fragment (annealing between o2227 and o2228) in NotI/EcoRI digested p9935
pSWVCR$_{126*}$-60bp-VCR$_{126**}$ (attP+)	pA702	**FRT** fragment in NotI/EcoRI digested p9936
pSW*attC*$_{aadA7}$-60bp-VCR$_{126}$ (attP-)	pA672	**FRT** fragment in NotI/EcoRI digested p9938
pSWVCR$_{126*}$-60bp-VCR$_{126**}$ (attP-)	pA671	**FRT** fragment in NotI/EcoRI digested p9937
pSW*attC*$_{aadA7}$-60+**3000bp*lacZ***-VCR$_{126}$ (attP+)	pA705	**FRT** fragment in NotI/EcoRI digested p8456
pSWVCR$_{126*}$-60+**3000bp*lacZ***-VCR$_{126**}$ (attP+)	pA697	**FRT** fragment in NotI/EcoRI digested p8459
pSW*attC*$_{aadA7}$-60+**3000bp*lacZ***-VCR$_{126}$ (attP-)	pA670	**FRT** fragment in NotI/EcoRI digested p8469
pSWVCR$_{126*}$-60+**3000bp*lacZ***-VCR$_{126**}$ (attP-)	pA663	**FRT** fragment in NotI/EcoRI digested p8467
pSW*attC*$_{aadA7}$-60+**750bp*lacZ***-VCR$_{126}$ (attP+)	pA696	**FRT** fragment in NotI/EcoRI digested p8458
pSWVCR$_{126*}$-60+**750bp*lacZ***-VCR$_{126**}$ (attP+)	pA717	**FRT** fragment in NotI/EcoRI digested p8460
pSW*attC*$_{aadA7}$-60+**750bp*lacZ***-VCR$_{126}$ (attP-)	pA669	**FRT** fragment in NotI/EcoRI digested p8463

pSWVCR$_{126*}$-60+**750bp*lacZ***-VCR$_{126**}$ (attP-)	pA667	**FRT** fragment in NotI/EcoRI digested p8461
pSW*attC*$_{aadA7}$-60+**1500bp*lacZ***-VCR$_{126}$ (attP+)	pA703	**FRT** fragment in NotI/EcoRI digested pA350
pSWVCR$_{126*}$-60+**1500bp*lacZ***-VCR$_{126**}$ (attP+)	pA701	**FRT** fragment in NotI/EcoRI digested pA351
pSW*attC*$_{aadA7}$-60+**1500bp*lacZ***-VCR$_{126}$ (attP-)	pA675	**FRT** fragment in NotI/EcoRI digested pA354
pSWVCR$_{126*}$-60+**1500bp*lacZ***-VCR$_{126**}$ (attP-)	pA673	**FRT** fragment in NotI/EcoRI digested pA352
pSW*attC*$_{aadA7}$-60bp-*attC*$_{ereA2}$ (attP+)	pB322	***attC*$_{ereA2}$** fragment (annealing between o2351, o2352, o2353 and o2354) in BamHI/SmaI digested pA621
pSW*attC*$_{aadA7}$-60bp-*attC*$_{ereA2}$ (attP-)	pB256	***attC*$_{ereA2}$** fragment in BamHI/SmaI digested pA672
pSW*attC*$_{aadA7}$-60+**3000bp*lacZ***- *attC*$_{ereA2}$ (attP+)	pB321	***attC*$_{ereA2}$** fragment in BamHI/SmaI digested pA705
pSW*attC*$_{aadA7}$-60+**3000bp*lacZ***- *attC*$_{ereA2}$ (attP-)	pB252	***attC*$_{ereA2}$** fragment in BamHI/SmaI digested pA670
pSW*attC*$_{aadA7}$-60+**750bp*lacZ***- *attC*$_{ereA2}$ (attP+)	pB254	***attC*$_{ereA2}$** fragment in BamHI/SmaI digested pA696
pSW*attC*$_{aadA7}$-60+**750bp*lacZ***- *attC*$_{ereA2}$ (attP-)	pB257	***attC*$_{ereA2}$** fragment in BamHI/SmaI digested pA669

pSW*attC*$_{aadA7}$-60+**1500bp*lacZ*-** *attC*$_{ereA2}$ (attP+)	pB255	***attC*$_{ereA2}$** fragment in BamHI/SmaI digested pA703
pSW*attC*$_{aadA7}$-60+**1500bp*lacZ*-** *attC*$_{ereA2}$ (attP-)	pB253	***attC*$_{ereA2}$** fragment in BamHI/SmaI digested pA675
pSW*attC*$_{aadA7}$-60+**400bp*lacZ*-**VCR$_{126}$ (attP+)	pB332	**400bp*lacZ*** fragment (PCR fragment (o863 and o870) from p923)) in BamHI/NheI digested pA696
pSWVCR$_{126*}$-60+**400bp*lacZ*-**VCR$_{126**}$ (attP+)	pB328	**400bp*lacZ*** fragment in BamHI/NheI digested pA717
pSW*attC*$_{aadA7}$-60+**400bp*lacZ*-** *attC*$_{ereA2}$ (attP+)	pB429	**400bp*lacZ*** fragment in BamHI/SmaI digested pB254
pSWVCR$_{126*}$-60+**400bp*lacZ*-**VCR$_{126**}$ (attP-)	pB327	**400bp*lacZ*** fragment in BamHI/NheI digested pA667
pSW*attC*$_{aadA7}$-60+**400bp*lacZ*-**VCR$_{126}$ (attP-)	pB331	**400bp*lacZ*** fragment in BamHI/NheI digested pA669
pSW*attC*$_{aadA7}$-60+**400bp*lacZ*-** *attC*$_{ereA2}$ (attP-)	pB428	**400bp*lacZ*** fragment in BamHI/SmaI digested pB257
pSW*attC*$_{aadA7}$-60+**750bp*hubP*-** *attC*$_{ereA2}$ (attP+)	pE511	**750bp*hubP*** fragment (PCR fragment (o3771 and o3773) from N16961 *Vibrio cholerae* (Heidelberg *et al*, 2000))) in BamHI/NheI digested pB255
pSW*attC*$_{aadA7}$-60+**750bp*hubP*-** VCR$_{126}$ (attP+)	pE507	**750bp*hubP*** fragment in BamHI/NheI digested pA703

pSW*attC*$_{aadA7}$-60+**750bp*hubP*-** VCR$_{126}$ (attP-)	pE503	**750bp*hubP*** fragment in BamHI/NheI digested pA675
pSW*attC*$_{aadA7}$-60+**1500bp*hubP*-** *attC*$_{ereA2}$ (attP+)	pE512	**1500bp*hubP*** fragment (PCR fragment (o3771 and o3774) from N16961 *Vibrio cholerae* (Heidelberg *et al*, 2000))) in BamHI/NheI digested pB255
pSW*attC*$_{aadA7}$-60+**1500bp*hubP*-** VCR$_{126}$ (attP+)	pE508	**1500bp*hubP*** fragment in BamHI/NheI digested pA703
pSW*attC*$_{aadA7}$-60+**1500bp*hubP*-** VCR$_{126}$ (attP-)	pE504	**1500bp*hubP*** fragment in BamHI/NheI digested pA675
pSW*attC*$_{aadA7}$-60+**3000bp*hubP*-** *attC*$_{ereA2}$ (attP+)	pE513	**3000bphubP** fragment (PCR fragment (o3771 and o3830) from N16961 *Vibrio cholerae* (Heidelberg *et al*, 2000))) in BamHI/NheI digested pB255
pSW*attC*$_{aadA7}$-60+**3000bp*hubP*-** VCR$_{126}$ (attP+)	pE509	**3000bp*hubP*** fragment in BamHI/NheI digested pA703
pSW*attC*$_{aadA7}$-60+**3000bp*hubP*-** VCR$_{126}$ (attP-)	pE505	**3000bp*hubP*** fragment in BamHI/NheI digested pA675
pSWVCR$_{16*}$-60-VCR$_{64}$ (attP+)	pE894	**VCR$_{16*}$-VCR$_{64}$** fragment (synthetic fragment) in NotI/NruI digested pA717
pSWVCR$_{16*}$-60-VCR$_{64}$ (attP-)	pE893	**VCR$_{16*}$-VCR$_{64}$** fragment in NotI/EcoRI digested pA667
pSWVCR$_{16*}$-60+**3000bp*lacZ*-** VCR$_{64}$	pE922	**3000bp*lacZ*** fragment in BamHI digested pE894

(attP+)		
pSWVCR$_{16*}$-60+**3000bp*lacZ*-** VCR$_{64}$ (attP-)	pE921	**3000bp*lacZ*** fragment in BamHI digested pE893
pSWVCR$_{16*}$-60+**400bp*lacZ*-** VCR$_{64}$ (attP+)	pE978	**400bp*lacZ*** fragment in BamHI digested pE922
pSWVCR$_{16*}$-60+**750bp*lacZ*-** VCR$_{64}$ (attP+)	pE979	**750bp*lacZ*** fragment in BamHI digested pE922
pSWVCR$_{16*}$-60+**1500bp*lacZ*-** VCR$_{64}$ (attP+)	pE980	**1500bp*lacZ*** fragment in BamHI digested pE922
pSWVCR$_{16*}$-60+**400bp*lacZ*-** VCR$_{64}$ (attP-)	pE975	**400bp*lacZ*** fragment in BamHI digested pE921
pSWVCR$_{16*}$-60+**750bp*lacZ*-** VCR$_{64}$ (attP-)	pE976	**750bp*lacZ*** fragment in BamHI digested pE921
pSWVCR$_{16*}$-60+**1500bp*lacZ*-** VCR$_{64}$ (attP-)	pE977	**1500bp*lacZ*** fragment in BamHI digested pE921

***** pSW plasmids were constructed in the Π1 strain growing with Thy supplemented in the medium. We confirmed plasmid constructions by performing PCR reactions and sequencing using o1897 and o1962 primers.

Table S1D

**Primers used in this study***

Primer name	Primer number	Sequences
RecReportF	o299	TTTGAATTCGCGGCCGCTTCTAGAGTGAGCGCAACGCAATTAATG
BamHIattCR	o845	AGTGGATCCCATCTAACGCTTGAATTAAGCCGCGC
5'BamHI-VCR-XmaI-1	o876	GATCCCATCTAACAAACGCCTCAAGAGGGACTGTCAACGCGTGGCGTTTCCAGTCCCATTGAGC
5'BamHI-VCR-XmaI-2	o877	CGCGGTGGTTGCTGTTGTTGTGTTTGAGTTTAGTGGTAGTGCGTTGTCAGCCCCTTAGGCGGGCGTTATAC
3'BamHI-VCR-XmaI-1	o878	GAAACGCCACGCGTTGACAGTCCCTCTTGAGGCGTTTGTTAGATGG
3'BamHI-VCR-XmaI-2	o879	CCGGGTATAACGCCCGCCTAAGGGGCTGACAACGCACTACCACTAAACTCAAACACAACAACAGCAACCACCGCGGCTCAATGGGACTG
5term	o883	GATCCAAAAAAAAACCCCGCCCCTGACAGGGCGGGGTTTTTTTTA
3term	o884	GATCTAAAAAAAACCCCGCCCTGTCAGGGGCGGGGTTTTTTTTG
modiVCR2-F	o885	AGGCGGGCGTTATTAC
modiVCR2-R	o886	CCGGGTAATAACGCCCGCCTAAG
5'BamHI-LacZ	o863	CGGGGATCCATGACCATGATTACGGATTCACTGG

3'NheI-BgLII-LacZ-3000	o864	GGCAGATCTGCTAGCCCAGGAGTCGTCGCCACCAATCC
3'NheI-LacZ-1500	o866	GGCGCTAGCAATAATATCGGTGGCCGTGGTGTCG
3'NheI-LacZ-750	o868	GGCGCTAGCCTCGCCGCACATCTGAACTTCAGC
3'NheI-LacZ-400	o870	GGCGCTAGCAGCTTTCATCAACATTAAATGTGAGC
XmaI-dapA-F	o1069	TTTCCCGGGTACTTCACGGGAAGTATTGT
PstI-dapA-R	o1070	CGGCTGCAGTTACAGCAAACCGGCATGCT
EcoRI-FRT-NotI	o2227	AATTCGAAGTTCCTATACtttctagaGAATAGGAACTTCGGAATAGG AACTTCGC
NotI-FRT-EcoRI	o2228	GGCCGCGAAGTTCCTATTCCGAAGTTCCTATTCtctagaaaGTATAG GAACTTCG
5BamHIereA2	o2351	GATCCAAAAAAAAACCCCGCCCCTGACAGGGCGGGGTTTTTTT TAGATCCCATCTAACC
5ereA2SmaI	o2352	TGCCAATCCACCGGACGGTTTTCAACCGCCGGTGATCAGCGCG TTATACCC
3ereA2SmaI	o2353	GGGTATAACGCGCTGATCACCGGCGGTTGAAAACCGTCCGGTG GATTGGCAGGTTAGATGGG
3BamHIereA2	o2354	ATCTAAAAAAAACCCCGCCCTGTCAGGGGCGGGGTTTTTTTTT

		G
5'-BamHI-*hubP*	o3771	CGGGGATCCCCAACGACTCCTGCTGCCAGTGGC
3'-750 NheI-*hubP*	o3773	GGCGCTAGCGAAGCTTCTTCCGCTTTACGGCGC
3'-1500 NheI-*hubP*	o3774	GGCGCTAGCGCCAGCTCATCATTGAGTAGCGTATCC
3'-3000 NheI-*hubP*	o3830	GGCGCTAGCACAATTCAGAAGGCGTACTTACTGCCATACCCGA GCGGAATCTTATTAAGCTCGGTAAAGGCTTCTTCCTCGGCTGG C
Swbeg	o1897	CCGTCACAGGTATTTATTCGGCG
Swend	o1898	CCTCACTAAAGGGAACAAAAGCTG
DAP-A-R	o1962	GTGGTGCCAACAGAAACGATCGC
Verif-lbd-attR	o361	GGCAAGCGCCTCGATTACTGCGATGTTTAG
finDAPrev	o3725bis	GGCCTTTCGTTTTATCTGTTGTTTGTCGG

**Synthetic fragments used in this study**

VCR$_{126*}$-term	in p8208	TCTAGAGTGAGCGCAACGCAATTAATGTGAGTTAGCTCACTCATTAGGCAC<br>CCCAGGCTTTACACTTTATGCTTCCGGCTCGTATGTTGTGTGGAATTGTGA<br>GCGGATAACAATTTCACACAGGAAACAGCTATGAATGATTACGAGTTCGGT<br>TATAACAAACGCCTCAAGAGGGACTGTCAACGCGTGGCGTTTCCAGTCCCA<br>TTGGCCGCGGTGGTTGCTGTTGTTGTGTTTGAGTTTAGTGGTAGTGCGTTG<br>TCAGCCCCTTAGGCGGGCGTTAGATGGGATCCAAAAAAAAACCCCGCCCCT<br>GACAGGGCGGGGTTTTTTTTAGATCT
VCR$_{16*}$-VCR$_{64}$	in pE884	GGCCCGCGGCCGCTTCTAGAGTGAGCGCAACGCAATTAATGTGAGTTAGCT<br>CACTCATTAGGCACCCCAGGCTTTACACTTTATGCTTCCGGCTCGTATGTT<br>GTGTGGAATTGTGAGCGGATAACAATTTCACACAGGAAACAGCTATGAATG<br>ATTACGAGTTCGGTTATAACAAACGGCTCAAGAGGGACTGTCAACGCGTGG<br>CGTTTCCAGTCCCAAGAACTGCGGCGTTTACGGTTGCTGTGTTTGAGTTTA<br>GTGTTAATGCGTTGCCAGCCCCTTAGCCGGGCGTTAGATGGGATCCAAAAA<br>AAAACCCCGCCCCTGACAGGGCGGGGTTTTTTTTAGATCCCATCTAACAAA<br>CGCCTTAAGAGGGACTGCCAACGCGTGGCATTTCCAGTCCCAATGAGCCGT<br>GGTGGTTACGGTTGTTGTGTTTGAGTTTCGTTGTTATGCGTTGTCAGCCCC<br>TTAGGCGGGCGTTATTACCCGGGTACTTCACGGGAAGTATTGTCGCGACCG<br>CG

***** Sequences are given in 5’→3’ direction.
